# Atomic‐Scale Insights into Nanoparticle Exsolution at Dislocations in Dislocation‐Engineered Catalysts

**DOI:** 10.1002/adma.202502362

**Published:** 2025-09-13

**Authors:** Moritz Lukas Weber, Moritz Kindelmann, Dylan Jennings, Jan Hölschke, Regina Dittmann, Joachim Mayer, Wolfgang Rheinheimer, Xufei Fang, Felix Gunkel

**Affiliations:** ^1^ Peter Grünberg Institute Electronic Materials (PGI‐7) Forschungszentrum Jülich GmbH 52425 Jülich Germany; ^2^ Next‐Generation Fuel Cell Research Center Kyushu University 744 Motooka Nishi‐ku Fukuoka 819‐0395 Japan; ^3^ Department of Materials Science and Engineering Massachusetts Institute of Technology Cambridge MA 02139 USA; ^4^ Institute of Energy Materials and Devices Materials Synthesis and Processing (IMD‐2) Forschungszentrum Jülich GmbH 52425 Jülich Germany; ^5^ Ernst Ruska‐Centre for Microscopy and Spectroscopy with Electrons (ER‐C) Forschungszentrum Jülich GmbH 52425 Jülich Germany; ^6^ Central Facility for Electron Microscopy (GFE) RWTH Aachen University 52064 Aachen Germany; ^7^ Institute for Manufacturing Technologies of Ceramic Components and Composites University of Stuttgart 70569 Stuttgart Germany; ^8^ Department of Materials and Earth Sciences Technical University of Darmstadt 64287 Darmstadt Germany; ^9^ Institute for Applied Materials Karlsruhe Institute of Technology 76131 Karlsruhe Germany; ^10^ Present address: Next‐Generation Fuel Cell Research Center Kyushu University Fukuoka, Japan & Department of Materials Science and Engineering Massachusetts Institute of Technology Cambridge MA USA; ^11^ Present address: DTU Energy Technical University of Denmark Lyngby Denmark; ^12^ Present address: Advanced Transmission Electron Microscopy Faculty of Physics and Astronomy Ruhr‐University‐Bochum Bochum Germany

**Keywords:** dislocations, dislocation engineering, epitaxial thin films, metal exsolution, nanoparticles

## Abstract

Achieving control over properties such as density and lateral distribution of catalytic nanoparticles under operation conditions is a major challenge for the development of active and durable catalysts, where nanoparticle coarsening is often the cause of performance degradation. While metal exsolution catalysts are regarded to be robust against this degradation mode, coarsening and increased concentrations of exsolved metal nanoparticles have been detected near extended defects. The present study examines the role of dislocations in metal exsolution reactions and explores the potential of dislocation‐engineering for the synthesis of dislocation‐associated nanoparticles. An atomic‐level correlation between bulk dislocations and surface nanoparticle locations is demonstrated through a novel approach for engineering epitaxial thin films with confined regions of increased dislocation densities in combination with in situ scanning transmission electron microscopy. While nanoparticle exsolution proceeds across the entire sample, two primary reasons for the frequent nucleation of dislocation‐associated nanoparticles are identified: the accumulation of exsolution‐active acceptors along dislocations and lattice distortions that are likely to lower the energy barrier for nanoparticle nucleation. This work establishes a proof of concept for using engineered dislocations in exsolution catalysts to synthesize nanoparticles with modified nanoparticle‐support properties relevant for the thermal stability and the lateral distribution of exsolved nanoparticles.

## Graphical abstract

This study reveals an atomic‐level correlation between bulk dislocations and the exsolution of surface nanoparticles through a novel approach for dislocation engineering in epitaxial thin films. Employing in situ scanning transmission electron microscopy, two primary reasons for the frequent nucleation of dislocation‐associated nanoparticles are identified: the accumulation of exsolution‐active acceptors along dislocations and lattice distortions induced by the dislocation structure.

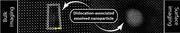

## 1 Introduction

Defect‐engineering of oxides lies at the heart of materials development for green energy technologies, for instance, where improved defect transport is required in energy conversion devices. The oxides’ defect structure typically defines its functional properties, where the precise control of the defect type(s) and concentration(s) plays a major role in the development of materials with advanced mechanical properties,^[^ ^1^, ^2^ ^]^ catalytic activity,^[^ ^3^, ^4^ ^]^ thermal conductivity^[^ ^5^ ^]^ or electron/ion conductivity.^[^ ^6^, ^7^, ^8^, ^9^, ^10^ ^]^ Here, chemical doping, control of the oxides’ microstructure, mechanical processing or advanced sintering strategies of functional materials are tools used to manipulate defect‐related properties of functional materials. Importantly, in oxides, the presence of defects of different dimensionality is typically intertwined. For instance, bulk point defect (0D) concentrations will considerably differ in close vicinity to defects of higher dimension such as dislocations (1D), surfaces,^[^ ^11^, ^12^, ^13^ ^]^ interfaces^[^ ^14^ ^]^ and grain boundaries^[^ ^15^, ^16^, ^17^, ^18^, ^19^, ^20^ ^]^ (2D). Furthermore, large concentrations of point defects may result in the formation of point defect clusters^[^ ^21^, ^22^, ^23^ ^]^ and ultimately in the nucleation of 3D inclusions of separated phases.^[^ ^24^, ^25^, ^26^, ^27^ ^]^

Moreover, defects are crucial for metal exsolution reactions, where the redox instability of metal dopants is exploited for the synthesis of metal‐oxide nano‐composite catalysts. Under thermally reducing conditions, the change in Gibbs free energy Δ*G* becomes negative for the metal oxide to metal transition, allowing for the fabrication of oxide‐supported metal nanoparticles, highly relevant for the application in key technologies for carbon‐neutral energy conversion, such as solid oxide fuel cells and electrolyzers.^[^ ^28^, ^29^ ^]^ Here, the defect structure impacts the energetics^[^ ^30^, ^31^ ^]^ and kinetics^[^ ^32^, ^33^ ^]^ of metal exsolution reactions as well as the nucleation^[^ ^34^, ^35^, ^36^ ^]^ and coalescence^[^ ^35^, ^37^ ^]^ of exsolved nanoparticles.

To date, the manipulation of 0D point defects – typically by chemical doping – is the most common strategy to tune the functionality of oxides. However, the concept of dislocation‐engineering, i.e., the introduction of 1D dislocations into materials to manipulate the functional properties of materials has been rising in interest in recent years.^[^ ^38^ ^]^ Here, the introduction of dislocations into exsolution‐active oxides may constitute an alternative route to locally alter material properties and to control the nanoparticle exsolution behavior. The role of dislocations in exsolution reactions has been so far unexplored, except from a very recent example pointing toward the nucleation of dislocations in a host oxide of limited redox‐stability during thermal reduction and a potential interplay between the dislocations with exsolution‐active Ru dopants.^[^ ^39^ ^]^

In this study, we investigate the impact of dislocations that are pre‐engineered into a redox‐stable oxide host on the exsolution behavior of reducible Ni dopants. While under thermal reduction, nanoparticle exsolution proceeds across the entire sample, i.e., in the presence and in the absence of dislocations, a considerable share of the synthesized surface nanoparticles is detected to be directly associated to dislocations. We identify that the accumulation of exsolution active acceptor dopants in the vicinity to the positively charged dislocation core is involved in providing an exsolution pathway alternative to metal exsolution from the coherent oxide lattice. Furthermore, we observe diminished surface dynamics of nanoparticles that nucleate at the engineered dislocation sites, which may be attributed to a structural stabilization via local lattice distortions and correlated strain fields. We expect that a lower energy barrier for particle nucleation at the dislocation contributes to the effect since severe lattice distortions are detected in the vicinity of the engineered dislocations. In all, our findings provide atomistic insights into the origin of frequent observations of preferential nanoparticle exsolution at extended defect structures such as grain boundaries. Moreover, our work demonstrates that dislocation‐engineering may be a promising strategy for tuning nanoparticle‐support properties of exsolved nanoparticles, which may hold potential to mitigate nanoparticle coarsening and to control catalyst properties such as nanoparticle density or nanoparticle distribution.

## 2 Results and Discussion

### 2.1 Dislocation Engineering of Oxide Thin Films by Epitaxy

**Figure 1:**
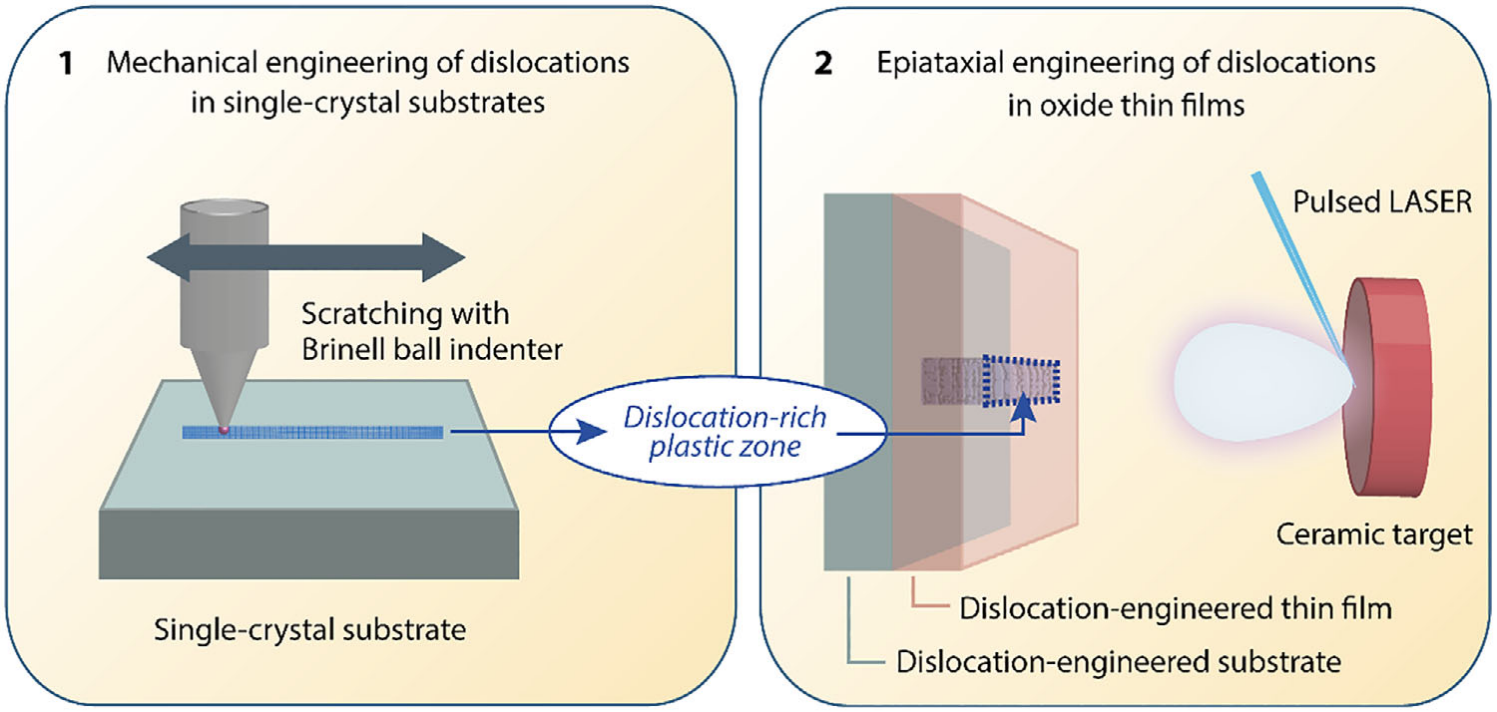
Schematic illustration of the experimental approach. Engineering of dislocation‐rich zones in epitaxially deposited functional oxides by pulsed laser deposition on single‐crystal substrates with mechanically induced dislocation‐rich plastic zones. 1) Dislocation‐rich plastic zones are mechanically induced into single‐crystal substrates by room‐temperature scratching. 2) Epitaxial oxide thin films are deposited by pulsed laser deposition, where dislocation‐rich zones are introduced from the substrate into the perovskite thin film. The dislocations introduced into the substrate serve as a template for the dislocations to further thread into the thin film during epitaxial growth.

To investigate the role of dislocations for metal exsolution reactions with high precision, a novel methodology for engineering dislocations into epitaxial thin films is developed (**Figure** **1**). In the first step, locally confined dislocation‐rich regions are mechanically imprinted into single‐crystal substrates, which are conventionally used for the synthesis of high‐quality epitaxial thin films. For this purpose, we use the room‐temperature Brinell indenter scratching method^[^ ^40^ ^]^ to generate laterally confined dislocation‐rich zones of ≈100 µm in width and depth, and several mm in length (cf. Figure S1, Supporting Information). In the second step, thin films are epitaxially grown on the dislocation‐engineered substrates by pulsed laser deposition (PLD). The dislocations in the substrate serve as nucleation sites^[^ ^41^ ^]^ to further transfer the dislocations into the epitaxial perovskite oxide layer.

**Figure 2:**
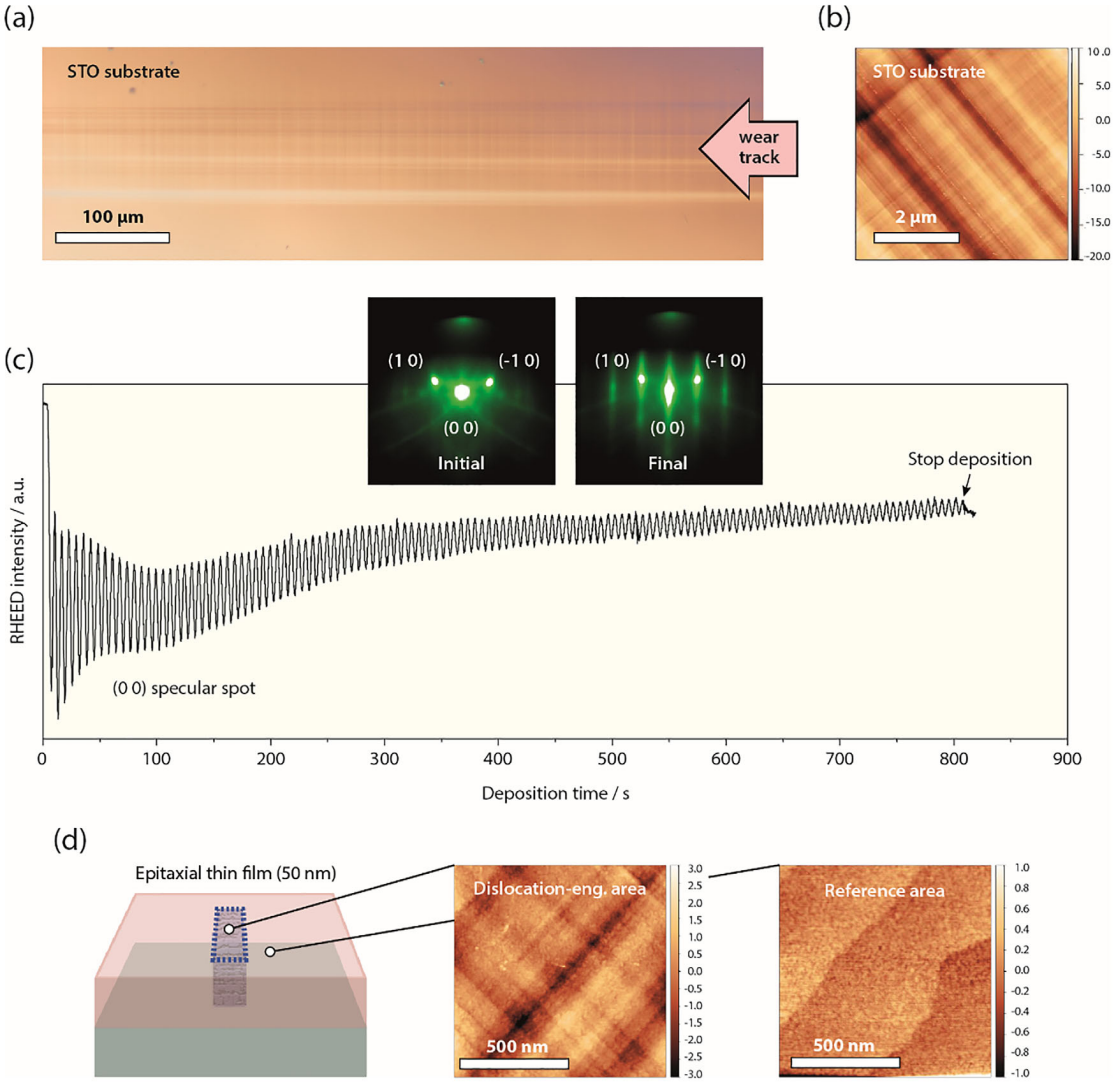
Mechanical plastic deformation of TiO_2_‐terminated SrTiO_3‐δ_ substrates and epitaxial growth of perovskite thin films with dislocation‐engineered zones. a) Representative optical microscopy imaging of a STO substrate after mechanical deformation, highlighting a wear track and b) atomic force microscopy image obtained within a wear track induced by a single pass via room‐temperature scratching. c) RHEED intensity evolution as monitored based on the (0 0) specular spot over the entire deposition time of a 50 nm thick STNi thin film, showing layer‐by‐layer growth. d) Schematic illustration of the obtained thin film sample with dislocation‐rich regions. Representative atomic force microscopy images are shown as obtained from a dislocation‐engineered region at the surface of a 50 nm thick STNi thin film and from a non‐engineered reference area of the same sample.

Following this approach, SrTi_0.95_Ni_0.05_O_3‐δ_ (STNi) thin films with defined dislocation‐rich regions are synthesized as depicted in **Figure** **2**. Dislocation‐rich zones were introduced into the TiO_2_‐terminated (001) single‐crystal SrTiO_3‐δ_ (STO) substrates with a 2.5 mm Brinell sapphire ball, applying a single scratching pass across its surface. Epitaxial growth of STNi on STO single‐crystal substrates enables the synthesis of thin films with only minor compressive strain of ≈ +0.1% introduced at the substrate‐to‐thin film interface (cf. Figure S2, Supporting Information). An influence of lattice strain on the exsolution behavior originating from the epitaxial relationship between the thin film and the substrate hence is expected to be negligible in the present material system.^[^ ^42^ ^]^ No lubricant or metallic Brinell ball material was used for the mechanical deformation process to avoid excessive contamination of the substrate (notably a minor Ca contamination was detected, cf. Figure S3, Supporting Information). Optical microscopy imaging shows a representative wear track along the (001) STO surface, revealing distinct slip traces indicating plastic deformation of the crystal, while no crack formation has been observed (Figure 2a).

As can be seen in Figure 2b, the respective slip traces furthermore become apparent by atomic force microscopy, where images are obtained within the extended region of the wear track (note that the scan angle is tilted ≈45° relative to the imaging direction of the optical microscopy image).

The dislocation‐engineered substrates are used for the growth of 50 nm thick STNi thin films by reflection high‐energy electron diffraction‐controlled pulsed laser deposition (RHEED‐PLD). Based on RHEED‐PLD the growth kinetics is monitored, tracking the evolution of the surface electron diffraction pattern over time. Here, continuous intensity oscillations are detected based on the (0 0) specular spot indicating a layer‐by‐layer growth mode for the entire growth duration (Figure 2c). On this basis, the deposition rate and the thin film thickness is controlled with monolayer precision. Importantly, the presence of dislocation‐engineered, i.e., highly defective regions at the surface of the single‐crystal substrate does not appear to hamper the applicability of RHEED monitoring. This may be possible since the RHEED pattern is recorded in grazing incidence toward the surface normal yielding a signal that is averaged across the surface, whereas the dislocation‐engineered zones are laterally confined.

Figure 2d shows a schematic illustration of the as‐deposited sample, where an engineered dislocation‐rich region is present in the epitaxial thin film, as induced during epitaxial growth of the perovskite oxide during RHEED‐PLD. As can be seen, the surface morphology of the thin film resembles the surface morphology observed for the mechanically deformed substrate (cf. Figure 2b), pointing toward a transfer of the defect structure into the functional oxide layer. In reference, AFM reveals a defined step terrace structure, when imaging a region of the thin film surface that nucleated atop a non‐deformed area of the substrate.

### 2.2 Atomic Structure of Dislocation‐Engineered Samples

**Figure 3:**
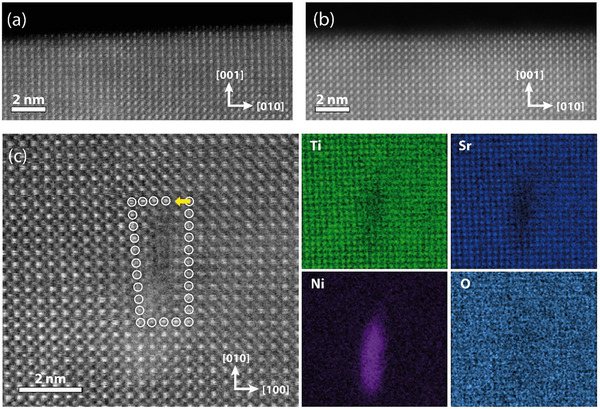
Representative ex situ characterization of an as‐grown SrTi_0.95_Ni_0.05_O_3‐δ_ thin film (a, b) in cross‐section geometry by STEM‐HAADF imaging and (c) plan‐view geometry by STEM‐HAADF imaging and EDXS mapping. a) High‐angle annular dark field imaging of the surface structure of an epitaxial thin film obtained from a plastically deformed area and b) a non‐deformed area for reference. c) Plan‐view HAADF‐STEM imaging reveals the presence of a dislocation with the Burgers vector b = 〈100〉 at the thin film surface. The Burgers circuit is indicated clockwise by encircled atoms, while the Burgers vector is denoted by a yellow arrow. EDXS mapping reveals the segregation of Ni‐acceptors in the vicinity of the dislocation core, and the depletion of Sr and Ti relative to the surrounding perovskite lattice.

**Figure** **3** presents a representative atomic‐scale analysis of an as‐deposited STNi thin film by high‐angle annular dark field (HAADF) imaging and energy dispersive x‐ray spectroscopy (EDXS). To unveil the structural features of the perovskite oxide lattice two different imaging geometries are used. Figure 3a,b depicts the atomic near‐surface structure of the STNi thin film comparing a dislocation‐rich region (Figure 3a) and a reference area (Figure 3b) detected in cross‐section geometry. Both the dislocation‐rich and the reference area exhibit a coherent perovskite lattice, where no significant differences in the defect structure are apparent. This is in line with expectations as 1D defects such as dislocations in an extended crystal lattice of tens of nanometers in thickness (FIB lamella) will only become visible by HAADF imaging when their orientation is favorable for the given imaging conditions (cf. Figure S3, Supporting Information).

In comparison, Figure 3c shows STEM‐HAADF imaging of the dislocation‐engineered region in plan‐view geometry. This approach enables imaging of the atomic structure perpendicular to the surface of the thin film sample. As can be seen, dislocations become apparent in the dislocation‐engineered area, as highlighted by the encircled atoms forming the respective Burgers circuit. 1D dislocations become visible using this imaging direction as they appear to be mostly aligned vertically between the substrate‐to‐thin film interface and the surface of the Ni‐doped STO thin film. Consequently, the defect is extended in imaging direction resulting in distinct contrast features. This interpretation of the plan‐view STEM‐HAADF imaging is confirmed by complementary weak‐beam dark‐field transmission electron microscopy (WBDF‐TEM) imaging providing direct evidence for dislocations threading from the substrate into the epitaxial thin film as presented in Figure S4 (Supporting Information). It is worth noting that by STEM‐HAADF imaging only edge components of dislocations are detectable, while screw components will remain elusive, as the atoms in these structural features are displaced in imaging direction, which is a common challenge in the microscopy of dislocations with screw components. Notably, the engineered dislocations present in our epitaxial thin film exhibit the same dislocation line direction but exhibit variety in their Burgers vectors as resolved by WBDF‐TEM (cf. Figure S4, Supporting Information).

Interestingly, EDXS mapping reveals a considerable enrichment of Ni‐acceptors in the vicinity to the dislocation core (Figure 3c). In contrast, the Sr and Ti signals appear to be decreased in the vicinity of the dislocation core relative to the surrounding perovskite lattice. Here, we determine an excess of Ni ∼ 54 ± 5 atoms/nm and a depletion of Sr ∼ −22 ± 2 atoms/nm as well as Ti ∼ −20 ± 2 atoms/nm following a standardless quantification of the element composition at the dislocation. The analysis provides a total excess number of atoms averaged across the length of the dislocation through the sample based on the EDXS maps shown in Figure 3c. While indications for Sr deficiency have been frequently observed for dislocations in SrTiO_3_ in the past^[^ ^43^, ^44^, ^45^ ^]^ and may contribute to the results, the decrease in the Sr and Ti signal is mainly caused by the considerable enrichment of Ni relative to the surrounding lattice. Therefore, the dislocation core may be off‐stoichiometric with respect to the nominal Sr/Ti ratio in STNi, while an additional relative decrease in both the Sr and Ti signal is caused by Ni enrichment.

The Ni signal appears to be off‐centered with respect to the dislocation core detected by HAADF imaging, which is due to the extended nature of the dislocation into the oxide bulk and minor deviation of its’ orientation relative to the imaging direction. This is also reflected by diffuse bright contrast visible toward the bottom left of the dislocation core in the HAADF image (Figure 3c), which indicates that the position of the dislocation core in the lamella migrates as it extends through the lamella. Furthermore, we expect a difference in the information depth in HAADF imaging. Although being a bulk‐sensitive imaging technique, surface‐near structures contribute strongly to the image contrast in high resolution HAADF‐STEM images, due to the very small depth of focus in this imaging mode, and the fact that the probe is focused to the top surface of the lamella during imaging. Therefore, the atomic resolution contrast tends to arise from the structure of the top several nanometers of the lamella, and features which are buried in the lamella will show more diffuse contrast as they are out of focus relative to the surface. EDXS, however, provides information that is averaged across the entire sample thickness.

Our finding of an increased Ni concentration close to the dislocation is in line with the fact that dislocation cores in acceptor‐doped STO are oxygen deficient^[^ ^44^, ^46^, ^47^ ^]^ and exhibit a positive core charge associated to the presence of oxygen vacancies. The accumulation of oxygen vacancies originates from the lower enthalpy of formation for oxygen vacancies near the dislocation core relative to the bulk,^[^ ^15^, ^17^, ^20^, ^48^ ^]^ where segregation of acceptors of relative negative charge may compensate the positive core charge.^[^ ^7^, ^11^, ^49^ ^]^ Furthermore, it was demonstrated that acceptor dopants exhibit a strong tendency to enrich at the dislocation cores in acceptor‐doped STO even at comparably small doping concentrations,^[^ ^50^ ^]^ where additional effects such as lattice strain that originates from the relative difference in size between the host cations and the dopant cations need to be taken into account.^[^ ^49^, ^51^ ^]^ Importantly, the strain field associated to dislocations is known to result in a considerable driving force for the segregation of dopants.^[^ ^52^, ^53^, ^54^ ^]^ The enrichment of reducible metals has been previously demonstrated to play a role for metal exsolution in the case of planar defects such as anti‐phase boundaries.^[^ ^33^ ^]^ Furthermore, heteroepitaxial interfaces aligned toward the oxide surface have been discussed as potential facile diffusion pathways in exsolution‐active host oxides.^[^ ^55^ ^]^ It is worth noting that for one of the investigated dislocations, a reconstructed NiO_x_ phase became visible that indicates Ni segregation beyond a mere relative increase in occupancy of perovskite lattice sites at the dislocation core (cf. Figure S5a–c, Supporting Information). The crystallization of a NiO_x_ nano‐phase points toward a significantly altered defect chemistry and structure of the dislocation core. However, typically no such NiO_x_ lattice was detected for the engineered dislocations (cf. Figure S5d, Supporting Information). In addition, it should be noted that epitaxial thin film samples constitute non‐equilibrium samples, where oxidizing annealing likely results in a further increase the respective concentration of Ni acceptors at the dislocation cores.^[^ ^32^, ^56^ ^]^

Moreover, at the given high doping level, Ni acceptors in STNi form to a certain extent nano‐scaled Ni‐rich clusters embedded within the perovskite oxide.^[^ ^24^, ^37^ ^]^ Therefore, occasionally, separated nanophases are present within the Ni‐doped STO matrix, however, typically linked to more significant Ni enrichment as compared to the engineered dislocations, while not related to the formation of dislocations in the thin films (cf. Figure S6, Supporting Information). Here, we observe that the NiO_x_ nanostructures are coherently embedded into the perovskite lattice^[^ ^24^ ^]^ and do not lead to a systematic formation of dislocations, as indicated by closed Burgers circuits, surrounding the embedded nanostructures (Figure S6b, Supporting Information). Similarly, clustering and segregation of dopants must be expected for many oxide ceramics, where a high density of internal interfaces (grain boundaries) are present, where dopants may be accommodated. We have recently discussed the effect of dopant clustering for the metal exsolution response in STNi^[^ ^35^ ^]^ and therefore, will not focus on this aspect in the present work. The high doping concentration of Ni in our material system may increase the tendency of Ni accumulation at dislocations, however, dopant segregation to grain boundaries of oxide ceramics is commonly observed also in dilute material systems as it is associated with the compensation of the positive core charge and strain fields at grain boundaries and is often of utmost importance for their functional properties.^[^ ^18^, ^50^, ^56^, ^57^ ^]^ The doping level of 5 at% of Ni at the B‐site applied in this study represents typical dopant concentrations used in exsolution‐active host oxides, likely being representative for a large share of exsolution catalysts.

### 2.3 In Situ Investigation of Dislocations as Nucleation Sites in Metal Exsolution Reactions

**Figure 4:**
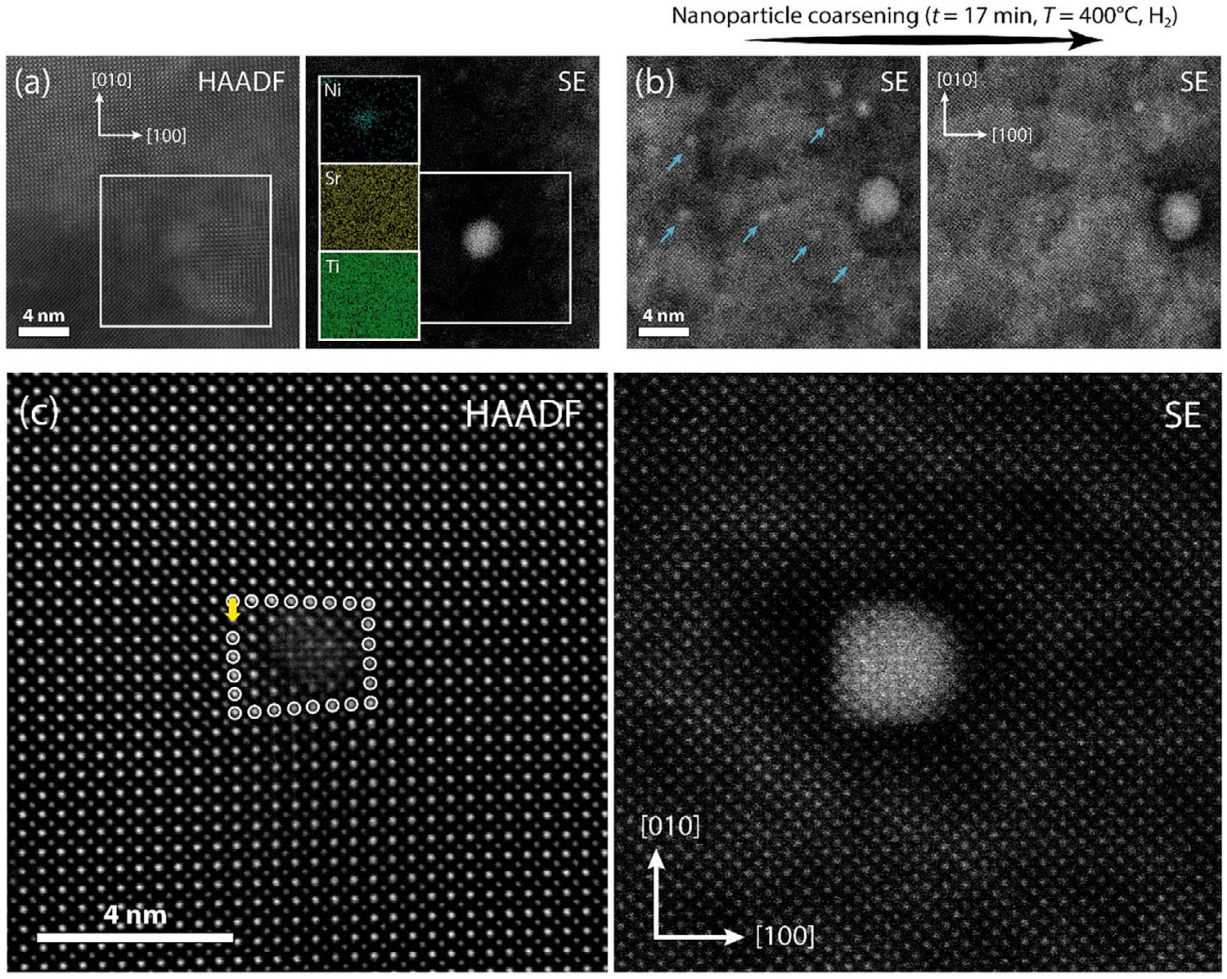
In situ analysis by correlated HAADF and SE imaging of nanoparticle exsolution during thermal reduction of a dislocation‐engineered SrTi_0.95_Ni_0.05_O_3‐δ_ thin film in plan‐view geometry using an environmental STEM. a) Detection of metal exsolution at T = 400 °C in hydrogen atmosphere from the Ni‐doped STO matrix with no dislocation present in the scan imaging area. EDXS analysis confirms the nucleation of nickel nanoparticles. b) SE imaging over the course of t = 17 min at T = 400 °C in hydrogen atmosphere reveals the coarsening of exsolved nanoparticles, where nanoparticles that appear to redissolve during Ostwald ripening or migrate forming nanoparticle clusters are highlighted by arrows. c) Correlated HAADF and SE imaging reveals the frequent formation of surface Ni nanoparticles at dislocations present in perovskite oxide matrix. A representative dislocation with a Burgers vector b = 〈100〉 is shown in the HAADF image. The Burgers circuit is indicated clockwise by encircled atoms and the Burgers vector is denoted by a yellow arrow. A Ni nanoparticle that exsolved at the dislocation core is detected by SE imaging. Images shown in (c) have been recorded at T = 600 °C in vacuum conditions.

To clarify the role of dislocations for Ni exsolution in STNi, environmental STEM studies have been conducted on the defect‐engineered STNi samples (**Figure** **4**). For this purpose, a plan‐view FIB lamella is mounted on a MEMS (micro‐electromechanical systems) chip allowing for sample heating in reactive gas environments. The environmental STEM employed in the current study enables simultaneous imaging of the bulk‐ and the surface structure by correlated HAADF and secondary electron (SE) image detection.^[^ ^35^ ^]^ Our methodology hence allows for the in situ analysis of the exsolution response of STNi, providing top‐view imaging of the oxide surface.

Figure 4a shows a region of the sample with no dislocations apparent by bulk‐sensitive HAADF imaging, and after heating to a temperature of *T* = 400 °C in a multi‐step procedure (*T*  =  300 °C in vacuum, *T* = 400 °C in vacuum, *T* = 400 °C in hydrogen). The correlated image recorded by the surface‐sensitive SE detector reveals an exsolved metal nanoparticle that nucleated from the perovskite matrix, i.e., in absence of a dislocation, where EDXS mapping confirms that the particle consists of Ni. Notably, first indications for the nucleation of nanoparticles were detected already after vacuum annealing at *T* = 300 °C (cf. Figure S7, Supporting Information), while annealing under hydrogen led to an increase in the nanoparticle density at the surface of the STNi sample. This observation may reflect an early exsolution onset relative to previous studies on samples without engineered dislocations, where first indications of nanoparticle exsolution have been detected at *T * =  400 °C under hydrogen atmosphere.

Subsequently, coarsening effects dominate the evolution in nanoparticle density resulting in a continuous decrease in the number of smaller nanoparticles, whereas other nanoparticles grow over the course of the thermal reduction treatment. Generally, thermally activated nanoparticle coarsening proceeds by two mechanisms, which are particle migration and coalescence as well as Ostwald ripening, referring to net ionic mass transport from smaller to larger nanoparticles. Nanoparticle coarsening is thermodynamically driven and rooted in the smaller average coordination of atoms in nanoparticles of decreasing size, which is associated with an increasing chemical potential.

Our investigations on dislocation‐engineered STNi reveals Ostwald ripening of exsolved nanoparticles to occur during thermal reduction, as visible from representative data presented in Figure 4b. Here, the same field‐of‐view of the STNi surface features a high density of small nanoparticles shortly after start of the reduction reaction, while a decreased number of nanoparticles is apparent after a time interval of 17 min under equal annealing conditions. Notably, none of the particles is detected to be associated to a dislocation, while a certain degree of lattice distortions is evident from corresponding HAADF imaging (cf. Figure S8, Supporting Information). During the investigations, no indications for nanoparticle migration were observed, implying that ionic mass transfer via Ostwald ripening plays the pre‐dominant role for nanoparticle coarsening in the dislocation‐engineered host oxide. In contrast, both Ostwald ripening and particle migration was detected for the same material, when no dislocation‐engineering was applied.^[^ ^35^ ^]^

After continuous thermal reduction with a stepwise increase of the reduction temperature up to a final temperature of *T* = 600 °C in vacuum, exsolved nanoparticles are investigated by correlated HAADF / SE imaging, where the location of a large share of the exsolved nanoparticles are detected to be associated to defects. It is worth noting that we have detected exsolved nanoparticles that are associated to dislocations already after reduction at *T* = 400 °C, while many images were recorded at *T* = 600 °C for in‐depth analysis after completion of the multi‐step annealing protocol. Figure 4c shows representative imaging of the correlated bulk and surface structure as obtained by in situ STEM, depicting an exsolved Ni nanoparticle that sits right at the center of an engineered dislocation (please refer to Figure S9 (Supporting Information) for more examples of high‐resolution images of dislocation‐associated exsolved nanoparticles). This observation is in line with the enrichment of Ni acceptors along the dislocation core, resulting in locally increased Ni concentrations in the subsurface of the oxide host, likely promoting the formation of exsolved nanoparticles at its surface. Moreover, nanoparticles sitting on the coherent perovskite surface, i.e., not associated to dislocations, as well as nanoparticles associated with nanocolumn defects^[^ ^35^ ^]^ have been detected.

After the thermal reduction procedure, images were recorded with lower magnification for a semi‐quantitative statistical evaluation of the nanoparticle population with respect to the nanoparticle‐support characteristics (cf. Figures S10–S15, Supporting Information). Here, we find that ≈10% of all investigated particles are associated with dislocations in the engineered samples. In view of the small local surface area modified by emerging dislocations (<1% of the total investigated surface area, considering an area of *A*∼9 nm^2^ disrupted per dislocation termination), this finding provides strong evidence that the formation of dislocation‐associated nanoparticles cannot be explained by a statistically random distribution of nucleation sites across the oxide surface. Rather, dislocations must be considered to serve as preferential nucleation sites in exsolution reactions. Moreover, this percentage provides only a lower limit of the real value, as our methodology likely underestimates the number of dislocation‐associated exsolved nanoparticles. The reason is that the imaging angle will allow only for the detection of the edge component of dislocations, but not the detection of screw dislocations or the screw components of mixed dislocations. This is especially relevant for the given dislocation‐engineered samples since the mechanically seeded dislocations via surface scratching are predominantly screw types intersecting the sample surface, which has been experimentally documented by the dislocation etch pit method^[^ ^58^ ^]^ and TEM analysis.^[^ ^59^, ^60^ ^]^ Notably, weak‐beam dark‐field TEM also revealed structurally variable dislocation types in the present dislocation‐engineered thin films (cf. Figure S4, Supporting Information).

Notably, significantly larger dislocation densities can in principle be introduced into the material (orders of magnitude^[^ ^40^ ^]^), by room‐temperature scratching, likely increasing the relative percentage of nanoparticles associated with dislocations. On the basis of the images provided in Figures S10–S15 (Supporting Information) we find that ≈60% of all investigated dislocations are associated to exsolved nanoparticles. Since HR‐STEM enables imaging of edge and mixed dislocations, but does not allow to further distinction between them, this finding may be consistent with recent results by Kim et al. suggesting that mixed dislocations play a particular role in exsolution reactions, while pure edge dislocations and pure screw dislocations may be less relevant to the process.^[^ ^39^ ^]^ Importantly, our semi‐quantitative evaluation provides only information based on a single sample state, i.e., a single moment in time on a surface that evolves dynamically due to simultaneous mass transfer of dopants toward the surface, nanoparticle nucleation and coarsening.^[^ ^35^, ^37^ ^]^ Therefore, it reflects solely a snapshot of the exsolved nanoparticle population at the time of detection rather than general characteristics of the material. Furthermore, a decrease in the concentration and mobility of oxygen vacancies in STNi due to a high density of dislocations with respective space‐charge tubes that are depleted from oxygen vacancies and surround the dislocation cores^[^ ^7^, ^61^ ^]^ needs to be considered. Given the fact that high oxygen vacancy concentrations at the nanoparticle‐support interface have been demonstrated to result in a decreased thermal stability of exsolved nanoparticles,^[^ ^37^ ^]^ the overlap of multiple space‐charge tubes with lower oxygen vacancy concentration may cause a decrease in the coarsening dynamics of exsolved nanoparticles in the dislocation‐engineered samples.

**Figure 5:**
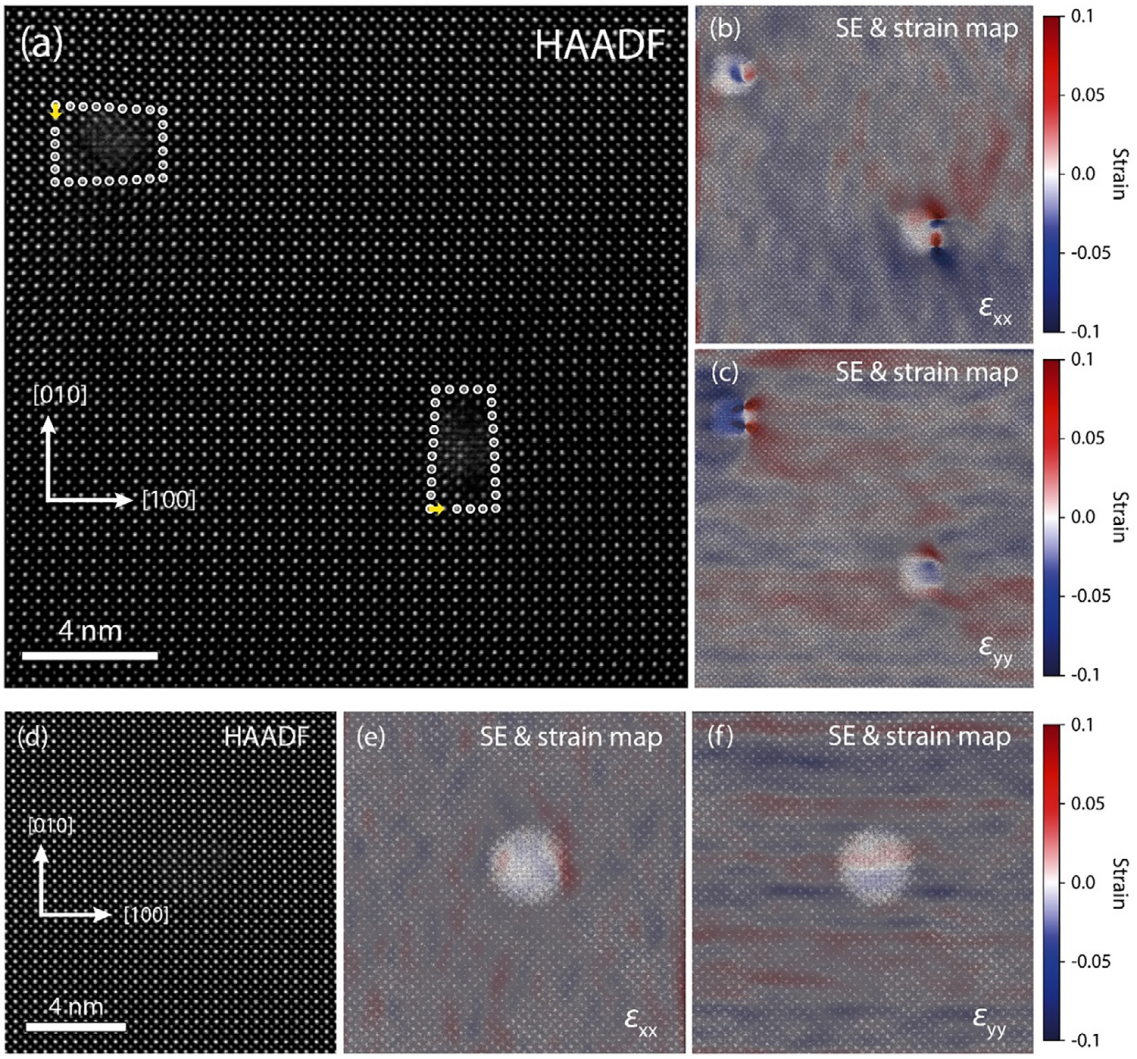
In situ investigation of nanoparticle exsolution during thermal reduction of a dislocation‐engineered SrTi_0.95_Ni_0.05_O_3‐δ_ thin film in plan‐view geometry by correlated HAADF and SE imaging using environmental STEM and strain mapping via geometric phase analysis. Dislocation‐associated exsolved nanoparticles and pristine nanoparticles are compared with respect to lattice strain at the nanoparticle‐support interface. a) Correlated HAADF/SE imaging of two exsolved nanoparticles associated with dislocations, respectively. The dislocations exhibit Burgers vectors of b = 〈100〉. The Burgers circuit is indicated clockwise by encircled atoms and the Burgers vectors are denoted by yellow arrows. b,c) SE images with superimposed strain maps obtained by geometric phase analysis reveal that significant strain fields are associated with the dislocation cores present in the oxide. d) Correlated HAADF/SE imaging of a single exsolved nanoparticle at the pristine perovskite surface with no dislocations present. e,f) SE images with superimposed strain maps reveal that no significant strain fields are present at the dislocation‐free nanoparticle‐support interface. Red color denotes tensile strain; blue color denotes compressive strain. All images have been recorded at T = 600 °C in vacuum conditions. The cut‐off strain level corresponding to the strain maps shown in (b, c, e, f) are −10% to +10%.

To get further insights into the structural properties at the nanoparticle‐support interface, geometric phase analysis is performed to investigate strain fields present in the perovskite lattice. **Figure** **5** illustrates HAADF images as well as corresponding SE images with superimposed strain maps visualizing the location of strain fields present in the oxide support with respect to the presence of engineered dislocations and dislocation‐associated exsolved nanoparticles (cf. Figure S16, Supporting Information for separate HAADF / SE images and strain maps). Two dislocations are apparent in Figure 5a, where dislocation‐associated nanoparticles are visible in the (identical) SE images in Figure 5b,c.

Geometric phase analysis of the corresponding high‐resolution STEM image, performed along the horizontal [100] lattice direction (ε_xx_) and the vertical [010] lattice direction (ε_yy_), allows to extract strain maps that are superimposed to the SE images, respectively. Here, the strain distribution is denoted by blue or red intensity indicating compressive strain or tensile strain present in the perovskite oxide lattice. As can be seen, both detected dislocations give rise to considerable strain fields in the oxide lattice, where tensile strain components and compressive strain components are detected in ε_xx_ direction (Figure 5b) and in ε_yy_ direction (Figure 5c). Moreover, two strain features are associated with each of the dislocations. This observation most likely indicates the early stages of dissociation of the dislocations^[^ ^62^ ^]^ into two partial dislocations allowing for the relaxation of strain at the dislocation core.^[^ ^63^ ^]^ Please also refer to Figure S17 (Supporting Information) for Fast‐Fourier transforms obtained in the dislocation areas shown in Figure 5a. In contrast, strain fields are not observed when dislocations are absent, i.e., for exsolved nanoparticles on the pristine perovskite surface or for nanoparticles associated with nanocolumn defects (Figure 5d–f). Notably, stripe‐like features in the strain maps are caused by artefacts arising from scanning instabilities during image collection (cf. Figure 5e,f).

Overall, our findings show that the frequent detection of dislocation‐associated nanoparticles during metal exsolution appears to be promoted by an accumulation of reducible dopants along the dislocations and diminished particle migration dynamics. Lattice distortions and correlated strain fields at the dislocations likely affect the nanoparticle mobility but may potentially also facilitate the nucleation. For instance, a considerable decrease in the energy barrier for nanoparticle nucleation at regions of increased lattice distortion may be expected.^[^ ^53^ ^]^ This finding may further indicate that the frequent formation of exsolved nanoparticles along grain boundaries^[^ ^64^, ^65^, ^66^, ^67^, ^68^, ^69^, ^70^, ^71^, ^72^, ^73^, ^74^ ^]^ and at extended defects^[^ ^33^, ^35^, ^36^ ^]^ not only originates from the accumulation of exsolution‐active cations in the vicinity of such defects, but further to be facilitated by lattice distortions. Here a potential locally decreased energy barrier for nucleation may result in a stabilizing effect of dislocations for nanoparticles under thermal reduction conditions.

Furthermore, oxygen vacancies, which are highly confined at the dislocation cores, are expected to result in a depletion of oxygen vacancies in the surroundings of the engineered dislocations due to electrostatic repulsion within the associated space charge regions. The emerging decrease in oxygen vacancy concentration and low oxygen ion mobility in the vicinity of the dislocations may be expected to result in a somewhat slower exsolution dynamics.^[^ ^37^, ^75^ ^]^ In contrast, however, we observed a low temperature onset of metal exsolution as compared to non‐dislocation‐engineered samples (cf. Figure S7, Supporting Information). This observation may indicate that a more facile ionic mass transfer or a lower nucleation barrier at surface defects promotes nanoparticle exsolution in dislocation‐engineered oxides. Moreover, it is worth noting that the depletion of oxygen vacancies in the surrounding of dislocations likely contributes to an increased thermal stability of dislocation‐associated nanoparticles.^[^ ^37^ ^]^

While it is commonly believed that cation diffusion along surfaces and interfaces is fast in comparison to bulk diffusion, De Souza has pointed out that studies on the diffusion of cations along dislocations in STO are sparse^[^ ^76^ ^]^ and only few indications for enhanced cation diffusivity associated with dislocations have been reported.^[^ ^77^ ^]^ Although the hypothesis of enhanced exsolution dynamics by Kim et al. along dislocations is intriguing, we believe that neither the data presented in their recent study^[^ ^39^ ^]^ nor the data presented in our present study fully confirms nor refutes this assumption. The observation of an early exsolution onset at mildly reducing conditions (*T* = 300 °C in vacuum) as compared to our previous work (*T* = 400 °C in hydrogen atmosphere) may in principle reflect enhanced nucleation rates in the dislocation‐engineered samples in comparison to non‐dislocation‐engineered samples. Given that the influence of dislocations on cation mass transport in strontium titanate is currently little understood, we suggest studies on the exsolution kinetics in dislocation‐engineered samples as future area of research to explore. Here, dislocation‐engineered model systems with defined probing geometry, may prove highly valuable in studying mass transport in exsolution‐active host oxides.

Moreover, it is worth noting that we have not detected the migration of dislocation‐associated exsolved nanoparticles or the redissolution of dislocation‐associated exsolved nanoparticles (as a part of Ostwald‐ripening). In contrast, coarsening of nanoparticles that exsolved at the pristine perovskite surface has been detected (cf. Figure 4b and reference^[^ ^35^ ^]^). This observation may suggest that dislocation‐associated nanoparticles exhibit an increased thermal stability relative to particles that exsolve at the pristine perovskite surface. The questions if faster exsolution along dislocations contributes to the frequent observation of larger nanoparticle densities at dislocations and along grain boundaries, or if the effect is merely caused by an increased thermal stability of dislocations‐associated nanoparticles remains to be elucidated in future experiments.

Since for one of the investigated dislocations, a reconstructed NiO_x_ phase became visible in the as‐prepared sample (cf. Figure S5a–d, Supporting Information), the exsolution of Ni nanoparticles from segregated Ni oxide nanophases may contribute to the exsolution response linked to specific engineered dislocations. The exsolution of Ni from Ni‐rich clusters must be expected to impact the energetics of exsolution reactions as release of Ni from the perovskite structure is known as a key energetic barrier in the reaction pathway^[^ ^30^ ^]^ and may further promote the formation of buried metal nanoparticles in addition to the buried nanoparticle population emerging via exsolution from the perovskite matrix.^[^ ^78^ ^]^ The precipitation of a NiO_x_ nanophase furthermore points toward a significantly altered defect chemistry and structure of the dislocation core that likely impacts the metal exsolution mechanisms coming into play under reducing reaction environments. However, typically no such NiO_x_ lattice was detected for the engineered dislocations (cf. Figure S5e, Supporting Information).

Our findings suggest that dislocation‐engineering of oxides may offer an opportunity for tuning nanoparticle properties in metal exsolution catalysts. Since lattice strain and electrostatic interactions, as the root cause for Ni enrichment along dislocation cores, are directly linked to the dislocation structure, our results may be transferable from the thin film model system scale to polycrystalline materials relevant for applications in green hydrogen based solid oxide cells.,^[^ ^2^, ^5^, ^79^ ^]^ while further research will be required to examine the potential of dislocation‐engineering for tailoring the functionality of exsolution materials. Dislocation‐engineering may be a lever to modify the thermal stability of nanostructured catalysts as well as nanoparticle‐support interactions with potential impact on the catalytic activity. However, secondary effects such as altered redox properties of the oxide host need to be considered for the overall performance of the functional composites. Therefore, future investigations are required to examine the impact of dislocation‐association of catalytic nanoparticles on the activity and stability of exsolution catalysts.

In view of the presented results, the common practice of polishing ceramic samples for the study of metal exsolution at smooth surfaces^[^ ^80^, ^81^, ^82^ ^]^ requires reconsideration, since it may significantly alter the original exsolution behavior by the introduction of high densities of ill‐defined near‐surface dislocations compared to the native surface of oxides.^[^ ^60^, ^83^ ^]^ Moreover, it should be noted that the methodology for engineering laterally confined dislocations into epitaxial thin films introduced in this paper holds potential to manipulate the properties of a broad range of thin film devices fabricated by epitaxial deposition. This concept might prove useful for optimizing the functionality of such devices or for studying structure‐functionality relationships.

## 3 Conclusion

We have explored dislocation‐engineering as a strategy to synthesize dislocation‐associated metal nanoparticles in exsolution catalysts, providing atomic‐level insights into nanoparticle exsolution at dislocations. To achieve this, we developed a novel method to introduce dislocations into perovskite oxide thin films during epitaxial growth, enabling the engineering of laterally confined regions with increased dislocation densities in well‐defined oxide samples. Through simultaneous in situ atomic‐resolution imaging of the catalyst's bulk and surface structure during thermal reduction, we demonstrate a clear correlation between dislocations penetrating the surface of the oxide host lattice and the presence of exsolved nanoparticles. The formation of dislocation‐associated exsolved nanoparticles is attributed to the accumulation of exsolution‐active dopants near the dislocation core, likely promoted by a combination of lattice strain and electrostatic space charge interactions, while significant Ni enrichment may occasionally lead to the crystallization of a Ni‐rich secondary phase along the dislocation linked to an altered defect chemistry and atomic structure. In addition, a decreased energy barrier for nanoparticle nucleation caused by the lattice distortions and associated strain fields induced by dislocations is expected to contribute to the phenomenon. Our study provides atomistic insights into the origin of nanoparticle exsolution at extended defect structures such as grain boundaries and establishes the proof of concept that dislocation‐engineering of exsolution‐active oxides may be useful to modify the exsolution behavior with the prospect of tuning nanoparticle densities and distributions of exsolved nanoparticles at oxide surfaces.

## 4 Experimental Section

### Engineering of Dislocations in SrTiO_3_ Single Crystal Substrates

Dislocation‐rich plastic zones are engineered into TiO_2_‐terminated (001) SrTiO_3_ substrates (Shinkosha Co., Ltd., Japan) by mechanical deformation via room‐temperature scratching as recently demonstrated.^[^ ^40^ ^]^ For this purpose, the backsides of the substrates were glued (Crystalbond 509‐3 T‐E‐Klebetechnik) to the sample holder of a hardness testing machine (Karl‐Frank GmbH, Germany). Here, the deformation was performed with a 2.5 mm Brinell sapphire ball by applying a single scratching pass with a load of 1 kg and a lateral scratching speed of 0.5 mm s^−1^. To avoid excessive contamination, no lubricant was used for the process. Unlike the hardened steel ball used in reference,^[^ ^40^ ^]^ the sapphire indenter used here excludes possible contamination of the substrate by metallic trace elements.

### Thin Film Growth

50 nm thick, epitaxial thin films were synthesized by PLD on TiO_2_‐terminated (001) SrTiO_3_ substrates with confined plastically deformed regions of engineered dislocation density. An excimer laser with a wavelength of λ = 248 nm was used for the ablation of the ceramic oxide target. The PLD target was synthesized by the Pechini method. The laser was operated with a repetition rate of *f * =  5Hz, and a laser fluence of *F* = 1.14 J cm^−2^. The backside temperature of the substrates was controlled by an IR‐laser to be *T* = 650 °C. The oxygen partial pressure was set to *p*(O_2_) = 0.108mbar. After the deposition, thin film samples were quenched to room temperature. The thin film growth was controlled using RHEED (kSA 400, k‐Space Associates Inc., USA).

### Substrate and Thin Film Characterization

The surface morphology of SrTiO_3_ substrates and STNi thin films was investigated by AFM imaging in tapping mode using a Nanosurf FlexAFM, (Nanosurf AG, Liestal, Switzerland) employing cantilevers with a tip curvature of <7 nm (Silicon‐ SPM‐Sensor, PPP‐NCHR‐20, Nanosensors, Neuchatel, Switzerland). Prior to mechanical deformation SrTiO_3‐δ_ were characterized using a Cypher AFM (Oxford Instruments Asylum Research Inc., Santa Barbara, USA). Optical microscopy (Zeiss Axio Imager2, Carl Zeiss AG, Oberkochen, Germany) was performed to image the surface slip traces in the plastically deformed regions of the SrTiO_3‐δ_ substrates using circularly polarized light–differential interference contrast mode (C‐DIC). The AFM image shown in Figure 2b was cut from a larger image to show details of the surface morphology. A polynomial background (second degree) was subtracted for AFM images obtained from the dislocation‐rich areas shown in Figure 2b,d to improve visibility of morphological features. High‐resolution X‐ray diffraction was employed to investigate the crystal structure of a 100 nm‐thick STNi thin film in 2Θ‐ω geometry (D8 Discover, Bruker, Karlsruhe, Germany).

### STEM‐HAADF and WBDF Imaging

A protective layer of carbon was sputtered onto the surface of each thin film to increase the conductivity of the sample prior to the preparation of electron transparent lamellae by focused ion beam (FIB). Cross‐section lamellae were cut from a representative location in the dislocation engineered and a non‐engineered reference area using focused ion beam SEM (FIB‐SEM, FEI Helios NanoLab 460F1, USA). The scanning transmission electron microscopy (STEM) was performed at 200 kV using a probe corrected Thermo Fischer Scientific (TFS) Spectra 300 microscope (Thermo Fischer Scientific, USA), which is equipped with a Super‐X EDS detector.

Weak beam dark field (WBDF) imaging was done utilizing a JEOL JEM‐2100 TEM. Two images were collected of the same location, one utilizing $\vec{g}$ =  [200] and one utilizing $\vec{g}$=  [020] for imaging, and the Burgers vectors of dislocations in the film were evaluated based using $\vec{g}\cdot \vec{b}$ analysis. Notably, the analysis utilizing only two g‐vectors is not comprehensive enough to fully determine the Burgers vector but provides insight into which dislocations have screw and edge character.

Plan‐view liftouts for ex‐ and in situ experiments were taken using a Thermo Fisher Scientific Helios NanoLab 460F1 FIB‐SEM from the dislocation‐engineered region of the STNi thin film, with a final milling energy of 2 kV. For in situ experiments, the plan‐view liftout was first thinned on a standard grid, then tilted 90° and transferred onto a MEMS chip. After MEMS‐chip attachment, the top surface of the lamella was polished with a final energy of 2 kV, to guarantee a clean surface for secondary electron imaging.

### Environmental STEM‐HAADF/SE Imaging

In situ STEM experiments were executed on plan‐view liftouts using a Hitachi High‐Technologies HF5000 environmental S/TEM, equipped with a secondary electron detector. In situ experiments utilized pure hydrogen, with gas pressures in the column expected to be in the range of 1–10 Pa. Ex situ and in situ plan‐view imaging was done at 200 kV. Beam currents during in situ measurements are expected to be in the range of 100–200 pA, depending on the experiment. EDS quantification was done using Hyperspy.^[^ ^84^ ^]^ Calculations of excess atoms at the dislocation were done according to a procedure established for grain boundary excess,^[^ ^85^ ^]^ with an additional geometrical factor included to account for the difference in dimensionality of the defect (1‐D versus 2‐D). Geometrical phase analysis on experimental images was performed utilizing a DigitalMicrograph plugin.^[^ ^86^ ^]^ The complete annealing procedure performed in the environmental STEM chamber involved consecutive steps of heating of the sample at *T*_1_ = 300 °C in vacuum, *T*_2_ = 400 °C for 1 h in hydrogen atmosphere, *T*_3_ = 500 °C for 20 min in hydrogen atmosphere and *T*_3_ = 600 °C for 2.5 h in vacuum.

### Statistical Analysis

To analyze the relative frequencies of nanoparticle‐support interface characteristics, contrast features are examined comparing secondary electron images, dark‐field images, bright‐field images and corresponding Fourier‐filtered images that were obtained from the same field‐of‐view at multiple imaging areas. For this purpose, six different sample regions have been investigated corresponding to a total investigated area of *A * =  54610 nm^2^ and a total of *n * =  154 exsolved nanoparticles. Here, nanoparticle locations are evident from the secondary electron images (bright contrast). Dislocations become visible by Fourier‐filtering, where lattice regions of low structural coherence appear as dark contrast features. Here, the percentage of exsolved nanoparticles that are located in close vicinity to the dislocations are assigned as dislocation‐associated nanoparticles (*n*_dislocation_ = 16). Ni‐enriched phase‐separated defects, so called nanocolumn defects, become visible in the dark‐field images as bright contrast features. The percentage of exsolved nanoparticles located in close vicinity to the nanocolumn defects are assigned as nanocolumn‐associated nanoparticles (*n*_nanocolumn _ =  64). Note that both nanocolumn defects that are associated with exsolved nanoparticles at the surface and nanocolumn defects that remain buried in the oxide bulk without being associated with exsolved nanoparticles at the oxide surface are visible in the bright‐field images. Nanoparticles that are located at the surface without a dislocation or nanocolumn defect present are denoted as pristine nanoparticles (*n*_pristine_ = 74). The errors represent the standard deviation (±SD) of the mean values.

## Conflict of Interest

The authors declare no conflict of interest.

## Author Contributions

M.L.W. and X.F. conceived and designed the experiments. M.L.W. synthesized the samples and characterized the samples by AFM and HR‐XRD. J.H. performed mechanical deformation of the single‐crystal substrates. M.K. performed ex situ STEM and EDXS analysis in cross section geometry. D.J. performed ex situ / in situ STEM studies and EDXS in plan‐view imaging geometry, WBDF analysis, as well as strain mapping by geometric phase analysis. M.K. and D.J. contributed equally to this work. M.L.W. evaluated the experimental data with contributions, including in‐depth discussions, from M.K., D.J., X.F., and F.G. R.D., J.M., W.R., X.F., and F.G. supervised the research. M.L.W. conceived and wrote the original manuscript and edited the manuscript with contributions from all authors. All authors reviewed the manuscript and have given approval to the final version of the manuscript. M.L.W., X.F., and F.G. jointly determined the research direction.

## Supporting information



Supporting Information

## Data Availability

The data that support the findings of this study are available in the supplementary material of this article.
