# Supplementary material for: Atomic‐Scale Insights into Nanoparticle Exsolution at Dislocations in Dislocation‐Engineered Catalysts
Source: Adv Mater. 2025 Sep 13;38(1):e02362. doi: 10.1002/adma.202502362 (PMC12759202; doi:10.1002/adma.202502362)
Supplement: Supplementary file 1 — Supporting Information [file ADMA-38-e02362-s001.docx]

Supporting Information

Atomic-Scale Insights into Nanoparticle Exsolution at Dislocations in Dislocation-Engineered Catalysts

Moritz Lukas Weber*, Moritz Kindelmann*, Dylan Jennings*, Jan Hölschke, Regina Dittmann, Joachim Mayer, Wolfgang Rheinheimer, Xufei Fang*, Felix Gunkel*\


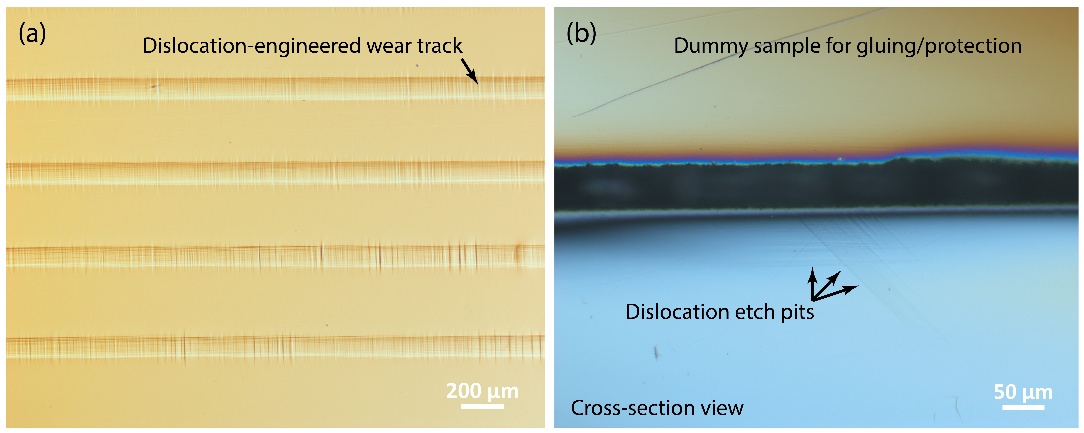


Figure S1. Investigation of the dimensions of plastically deformed areas in STO single-crystal substrates. (a) Top overview of the mm-long, ~100 μm wide wear tracks using Brinell scratching at room temperature on (001) STO substrate. (b) Cross-sectional view of the scratch track underneath the surface, showcasing the dislocations penerate about ~100 μm in depth. The top half is from a dummy sample, and the bottom part if the sample of interest. Note the features in Figure (a) are enhanced using the DIC mode in the optical microscope (Zeiss Axio Imager2) for better visualization. The local average roughness is less than 10 nm per 5 μm x 5 μm.

The dimensions of plastically deformed areas introduced into single-crystal STO substrates employed for thin film growth are illustrated in Figure S1.


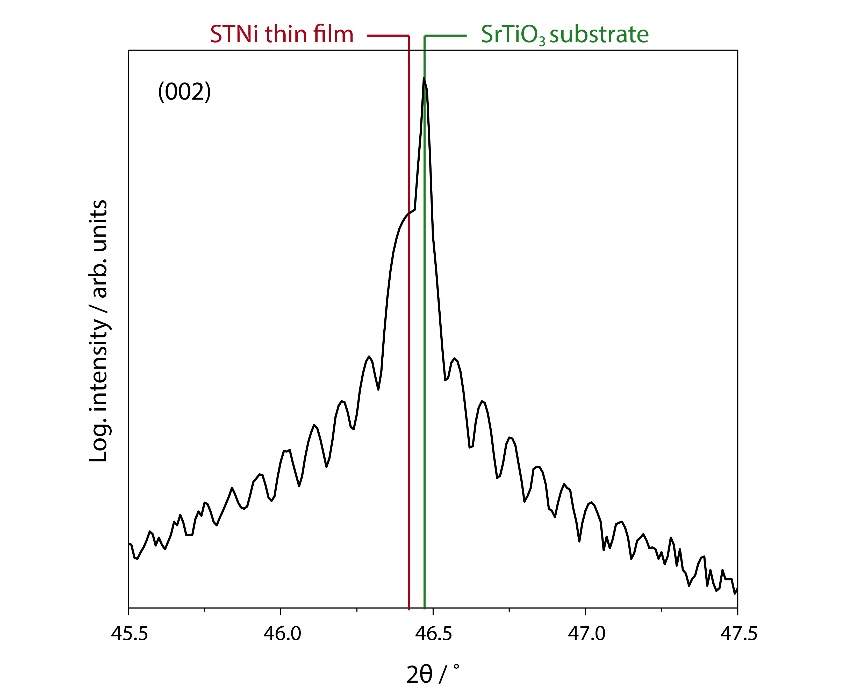


Figure S2. X-ray diffraction analysis obtained from a 100 nm thick STNi thin film deposited on a SrTiO_3‑δ_ single-crystal substrate. The (002) diffraction signals have been analyzed in 2θ-ω measurement geometry.

In the present study, SrTiO_3_ (STO) was chosen as substrate material, as it exhibits only a small difference in the lattice parameter to STNi resulting in only minor compressive strain of about +0.1 % induced by the epitaxial relationship. This becomes evident from X-ray diffraction analysis shown in Figure S2, where the diffraction signals originating from the thin film and from the STO substrate are detected at very similar diffraction angles indicating a similar lattice parameter of the materials. Due to a slightly larger lattice parameter of the thin film material, a shoulder at lower diffraction angles becomes visible.


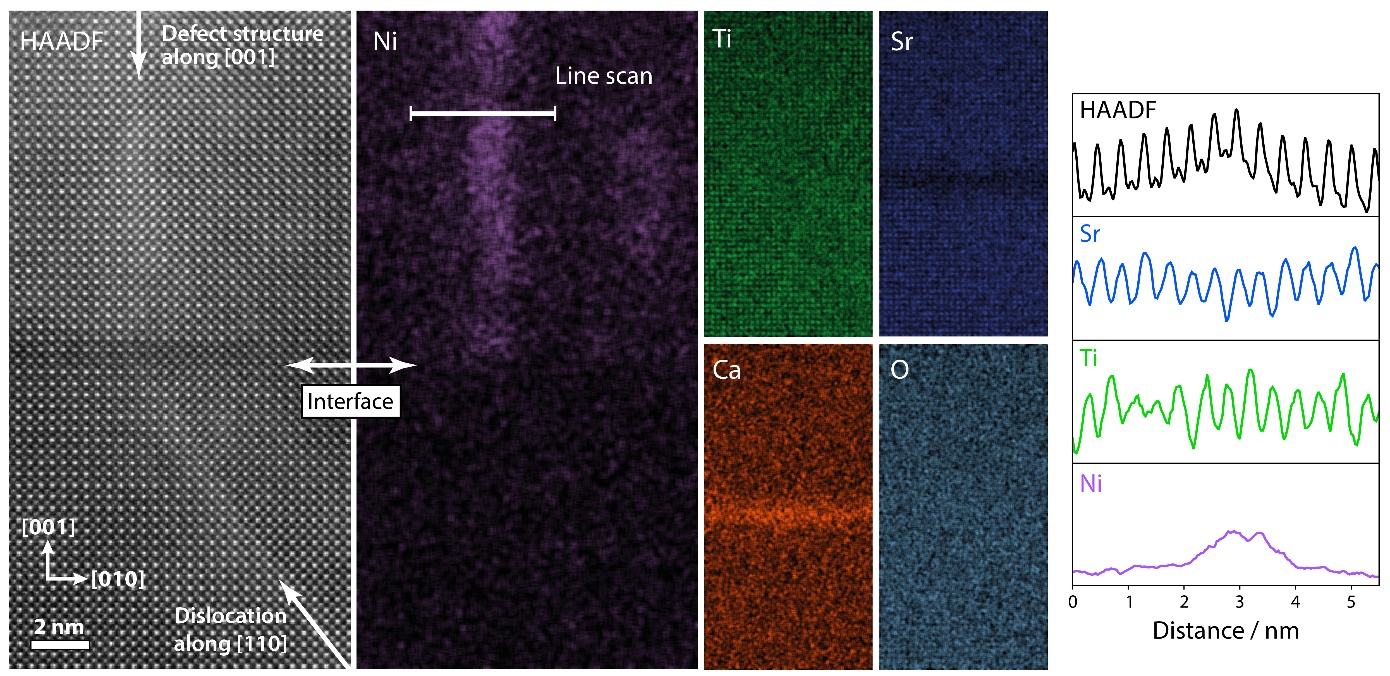


*Figure S3. Ex-situ STEM-HAADF imaging and EDXS mapping of an as-grown SrTi_0.95_Ni_0.05_O_3-δ_ thin film in cross-section geometry. The thin-film-to-substrate interface region of a dislocation-engineered region is shown. A dislocation present in the STO substrate and an extended defect structure associated with the dislocation at the interface as incorporated into the thin film during epitaxial growth is denoted by arrows. EDXS shows that the bright contrast detected in the oxide thin film corresponds to regions of increased Ni content. Lines profiles extracted from the HAADF image and EDXS maps is shown. Ca contamination is detected at the thin-film-to-substrate interface potentially originating from mechanical scratching of the surface using a sapphire indenter.*

Investigations of the substrate-to-thin-film interface by STEM-HAADF presented in Figure S2 provide further details on the defect structure of a sample obtained from a dislocation-rich area. A faint contrast feature becomes visible in the substrate region, likely indicating the presence of a dislocation oriented along the [110] direction of the crystal lattice. Interestingly, the presence of an extended defect structure in the STNi thin film can be inferred by a faint contrast feature that appears right above the point where the [110] dislocation penetrates the surface of the single-crystal substrate (see also results obtained by weak-beam dark-field imaging presented in Figure S3).The defect structure is oriented in [001] orientation, and, by means of EDXS analysis an enrichment of Ni acceptors is detected within the column-like defect.

As can be seen the line profile extracted from the HAADF image features intensity oscillations correlated to the atomic columns of the oxide lattice. Furthermore, an increase in the background intensity towards the center of the defect is visible that is related to the larger *Z*-contrast caused by the enrichment of Ni atoms. However, neither the Sr nor the Ti signal allows to make a clear statement with respect to strontium or titanium depletion, while a slight enrichment of Ni is detected. This is due to the cross-section imaging geometry, where line defects are embedded in a ~100 nm thick lamella, posing a considerable challenge for quantifying compositional changes. Notably, plan-view imaging enables a more accurate analysis of relative differences in the cation composition, as discussed in our original manuscript. Here, an excess of Ni ~ 54 ± 5 atoms/nm and a depletion of Sr ~ -22 ± 2 atoms/nm as well as Ti ~ -20 ± 2 atoms/nm following a standardless quantification of the element composition at the dislocation was determined. Notably, Ca contamination is visible at the substrate-to-thin film interface.


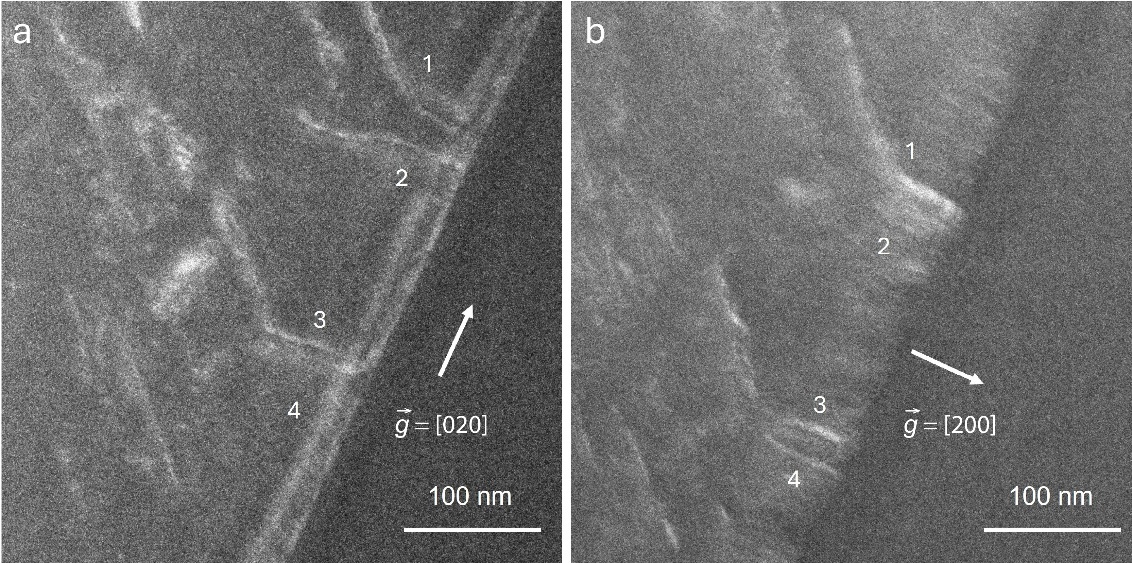


Figure S4. Weak-beam dark-field TEM analysis of a dislocation-engineered STNi sample. Dislocations become apparent in bright contrast, where imaging is performed using two different diffraction vectors g (a, b). A white arrow indicates the respective g vector used for imaging.

WBDF-TEM images obtained by using two different diffraction vectors *g* are shown in Figure S4a and Figure S4b, which correspond to in-contrast and out-of-contrast conditions, where dislocations become visible in one or in both images, depending on the Burgers vector. As can be seen, dislocations are present typically along the [110] slip planes in the substrate (left region of the images), and thread into the epitaxially deposited oxide thin film in the [001] direction (cf. HAADF-STEM analysis in Figure S3). In addition, $\vec{g}\cdot\vec{b}$ analysis allows us to specify the structural nature of the dislocations (1)-(4) visible, where dislocations (1) and (3) exhibit a mixed edge and screw character (e.g. $\vec{b}$ = [110] or $\vec{b}$ = [111]), dislocation (2) exhibit edge e.g. $\vec{b}$ = [010] or mixed e.g. $\vec{b}$ = [011] structure, while dislocation (4) exhibit screw e.g. $\vec{b}$ = [100] or mixed e.g. $\vec{b}$ = [101] character.


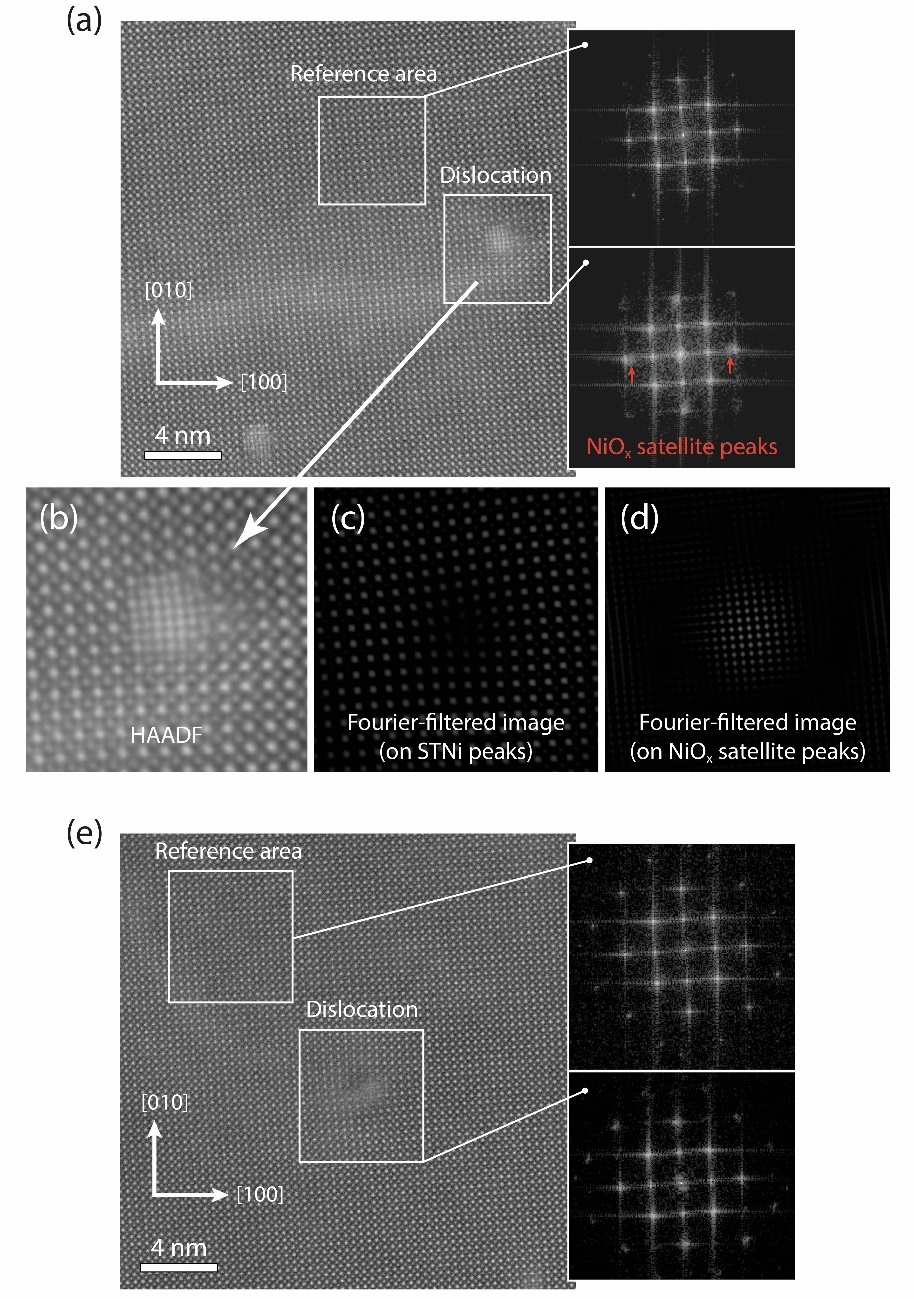


Figure S5. (a) Ex-situ STEM-HAADF imaging and Fast Fourier transforms obtained from different areas of the Z-contrast images. An engineered dislocation is compared to a reference area, where no dislocation is visible in the perovskite lattice, respectively. Faint satellite peaks are visible in the Fast Fourier transforms in the vicinity of the main diffraction peaks originating from a separated NiO_x_ phase present along the dislocation core. (b) A close-up of the separated NiOx phase present in the dislocation is shown below, where Fourier-filtering on the corresponding (c) main peaks and (d) satellite peaks of the separated phase confirm the presence of NiO_x_ coherently embedded in the STNi lattice. (e) Exemplary STEM-HAADF imaging and Fast Fourier transforms obtained from a dislocation area and reference area, where no NiO_x_ satellite peaks are detected in the dislocation. Notably, the case illustrated in (e) represents the commonly observed scenario.

Figure S5a shows an example of significant Ni enrichment along an engineered dislocation, where the presence of a distinct NiO_x_ phase becomes visible. The NiO_x_ nanophase is further evidenced in the Fast Fourier Transform of the HAADF image obtained from the dislocation region, appearing as faint satellite peaks (indicated by red arrows). A close-up view of the NiO_x_-enriched dislocation is shown below, where Fourier-filtered images reveal the STNi lattice (Figure S5b) and the NiO_x_ lattice (Figure S5c) more clearly.

However, in most cases, no distinct NiO_x_ phases are observed at the engineered dislocations (Figure S5d). This suggests either that Ni enrichment along the dislocations typically occurs without NiO_x_ nucleation (Ni remains integrated into the perovskite structure) or that a NiO_x_ phase is present but buried within the bulk of the sample, making it less detectable.


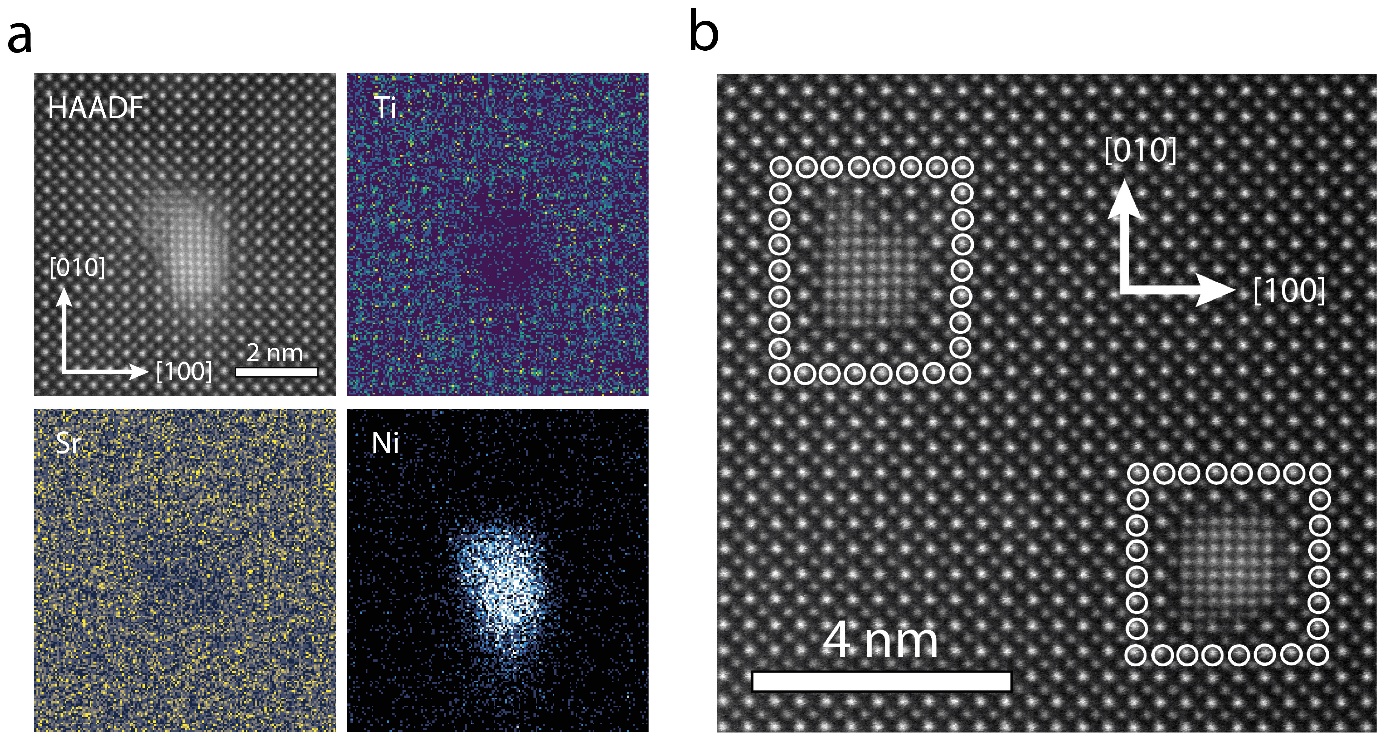


Figure S6. (a) Ex-situ STEM-HAADF imaging and EDXS mapping of an as-grown SrTi_0.95_Ni_0.05_O_3-δ_ thin film in plan-view geometry. A detailed image of a nanocolumn defect is shown, which exhibits enrichment of Ni dopants, while Sr and Ti are depleted. (b) The presence of nanocolumns is not accompanied by the presence of dislocations, which is evident from the Burgers circuits denoted by encircled atoms.

Nanocolumn defects occasionally form within the Ni-doped STO matrix^[35]^. An example STEM-HAADF image and corresponding EDXS analysis are shown in Figure S6a. Here, a NiO_x_ nanophase is coherently embedded in the perovskite structure where an excess of Ni ~ 48 ± 5 atoms/nm and a depletion of Sr ~ -69 ± 7 atoms/nm as well as Ti ~ -38 ± 4 atoms/nm is detected by a standardless quantification of the element composition. In comparison to the engineered dislocations, a stronger Sr and Ti depletion is detected. This may indicate that Ni is increasingly integrated into the engineered dislocation rather than replacing Sr and Ti sites with a separate nanophase. Burgers vectors for two representative embedded nanophases are denoted in Figure S6b, providing evidence that no systematic formation of dislocations is associated with Ni clustering. Rather, dislocations are solely detected in the thin film samples when dislocation-engineering of the substrate is performed as discussed in the main manuscript.

***
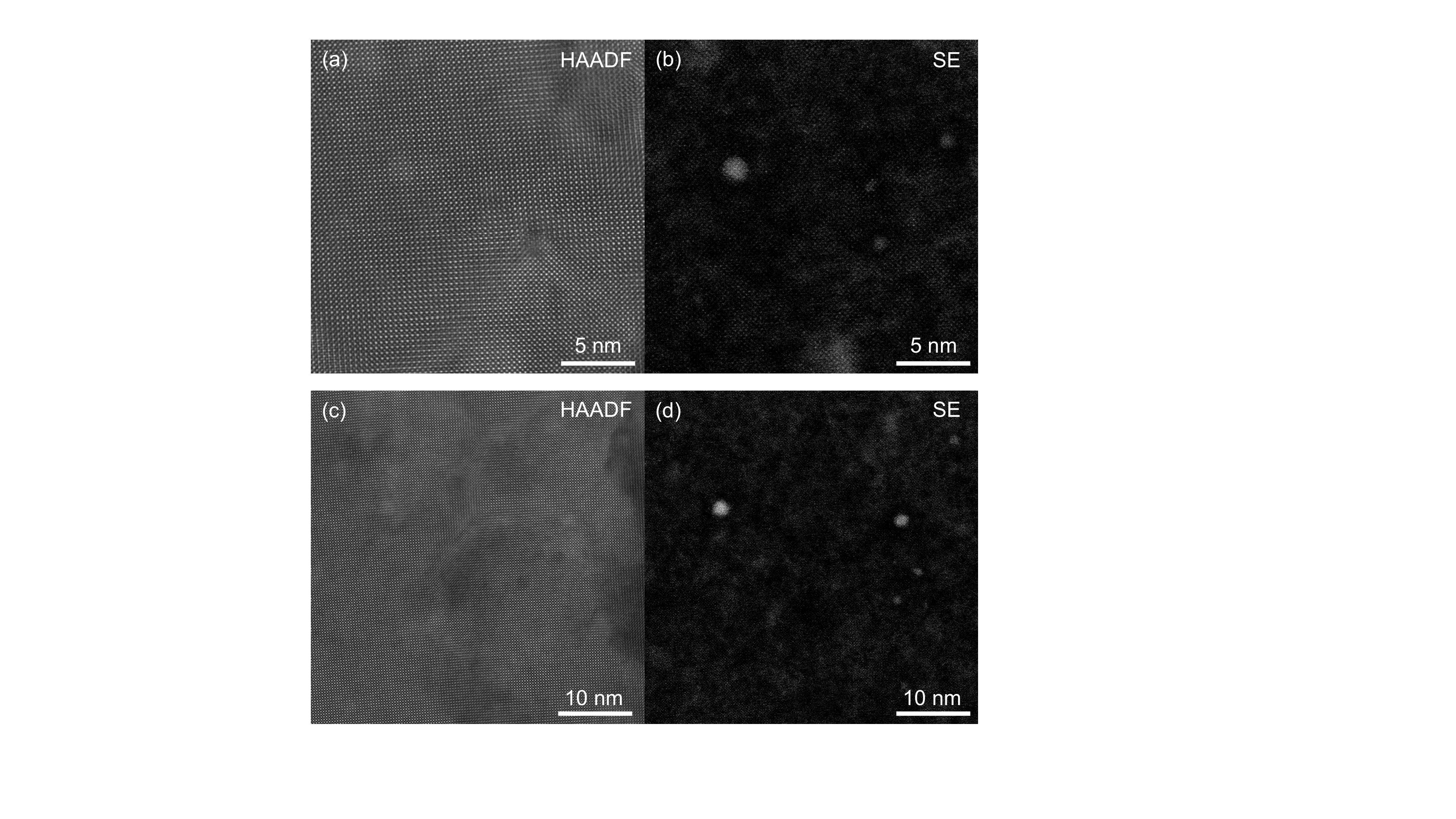
***

Figure S7. HAADF (a,c) and corresponding SE (b,d) images of the STNi sample with nanoparticles which had formed at T = 300°C under vacuum during in-situ experiments. Nanoparticles located above dislocations and away from dislocations are visible in the images. Exsolution was observed at lower temperatures than comparable in-situ experiments done with pristine thin films of the same composition (T = 300°C vs. T = 400°C).

An onset of nanoparticle exsolution in dislocation-engineered samples was detected at *T* = 300°C in vacuum. It is worth noting that this is a lower temperature than observed in previous in-situ experiments on non-dislocation-engineered thin films of the same material system ^[35]^.


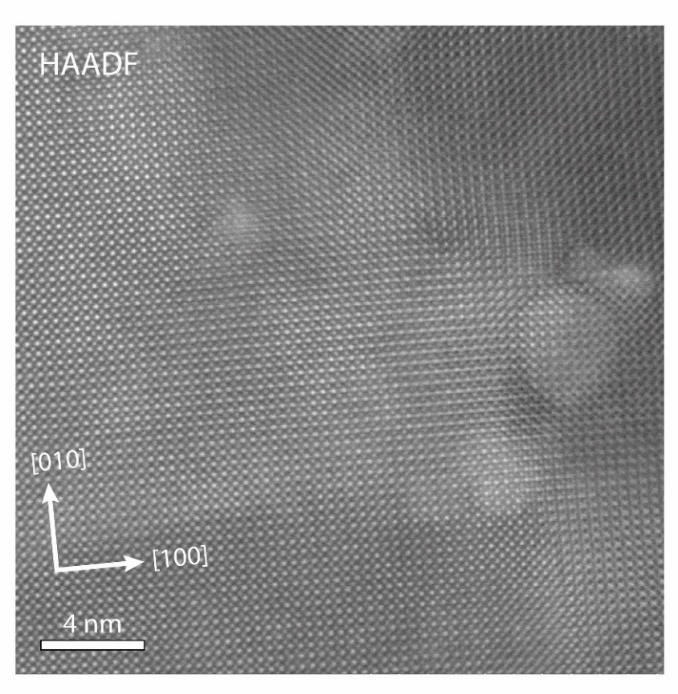


Figure S8. In-situ STEM-HAADF imaging of an as-grown SrTi_0.95_Ni_0.05_O_3-δ_ thin film in plan-view geometry corresponding to the field of view shown in Figure 4b of the main manuscript. While a certain degree of distortion is visible, particularly on the right-hand side of the HAADF image, no distinct edge dislocation is detected. Therefore, the exsolved particles shown in the secondary electron images (main manuscript, Figure 4b) are not associated with dislocations.


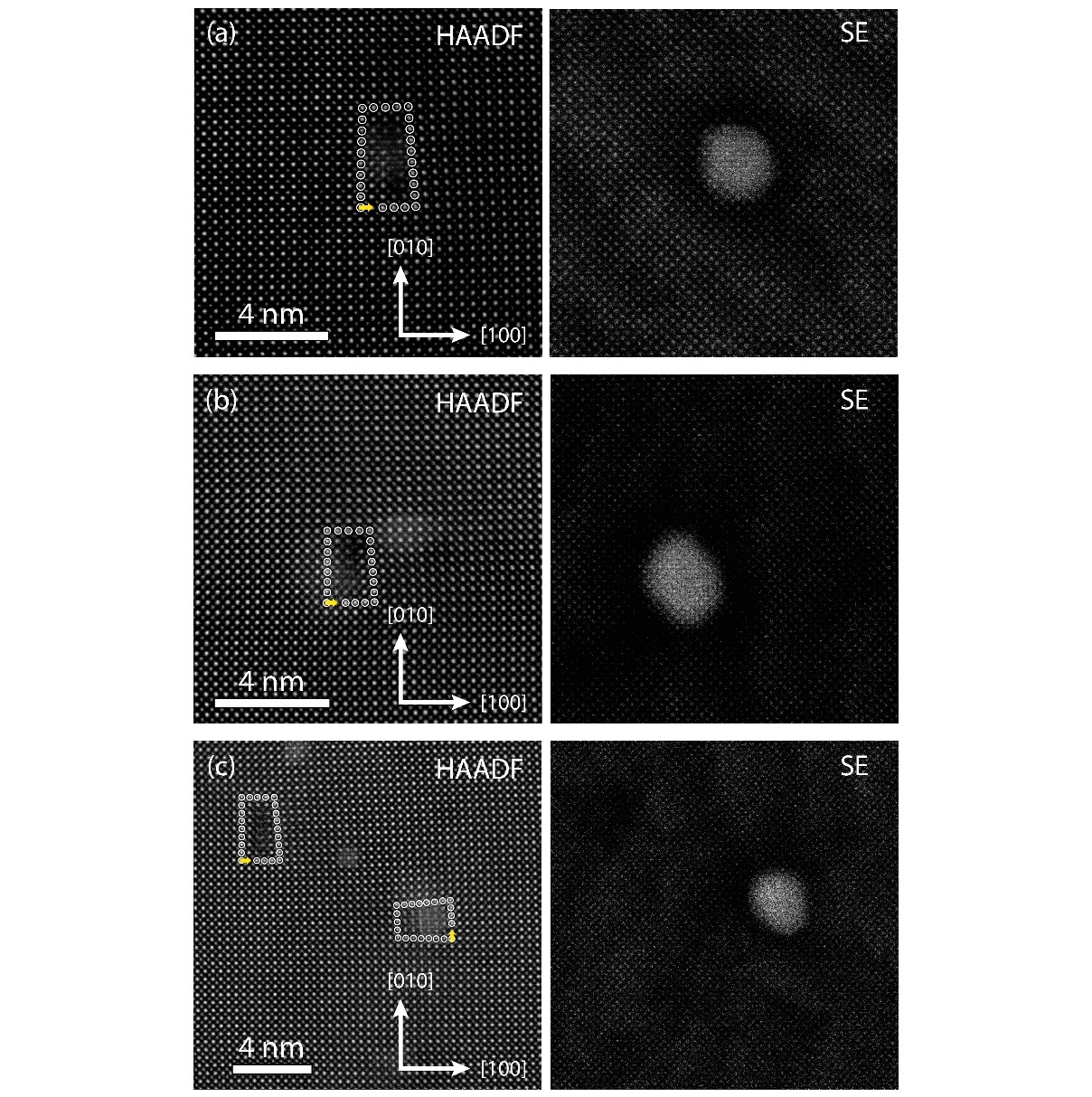


Figure S9. In-situ analysis by correlated HAADF and SE imaging of nanoparticle exsolution during thermal reduction of a dislocation-engineered SrTi_0.95_Ni_0.05_O_3-δ_ thin film in plan-view geometry using an environmental STEM. (a-c) Exemplary high-resolution images of nanoparticles exsolved at the perovskite surface and associated to dislocations.

We note that we observed a certain degree of structural variety in the engineered dislocations, e.g. with respect to termination planes present in the dislocation core. Further complexity is potentially caused by an early stage of grain boundary dissociation^[62]^, which is occurs due to large strain fields present at the dislocation to decrease the accumulated strain energy in the system (please see also strain maps in Figure S16). Moreover, mixed dislocations may be present in the sample, however, in-plane imaging geometry will not allow for the detection of screw components, while only edge components of the dislocations will become apparent. In addition, Ni atoms accumulated in the dislocation core results in images of the superimposed perovskite and NiO_x_/Ni sublattice, further increasing the perceived structural complexity. Notably, it appears that the shape of the nucleated nanoparticles oftentimes may deviate from the idealized circular or facetted shape, which might be related to the strain field associated to the dislocations present at the nanoparticle-support interface^[53]^.


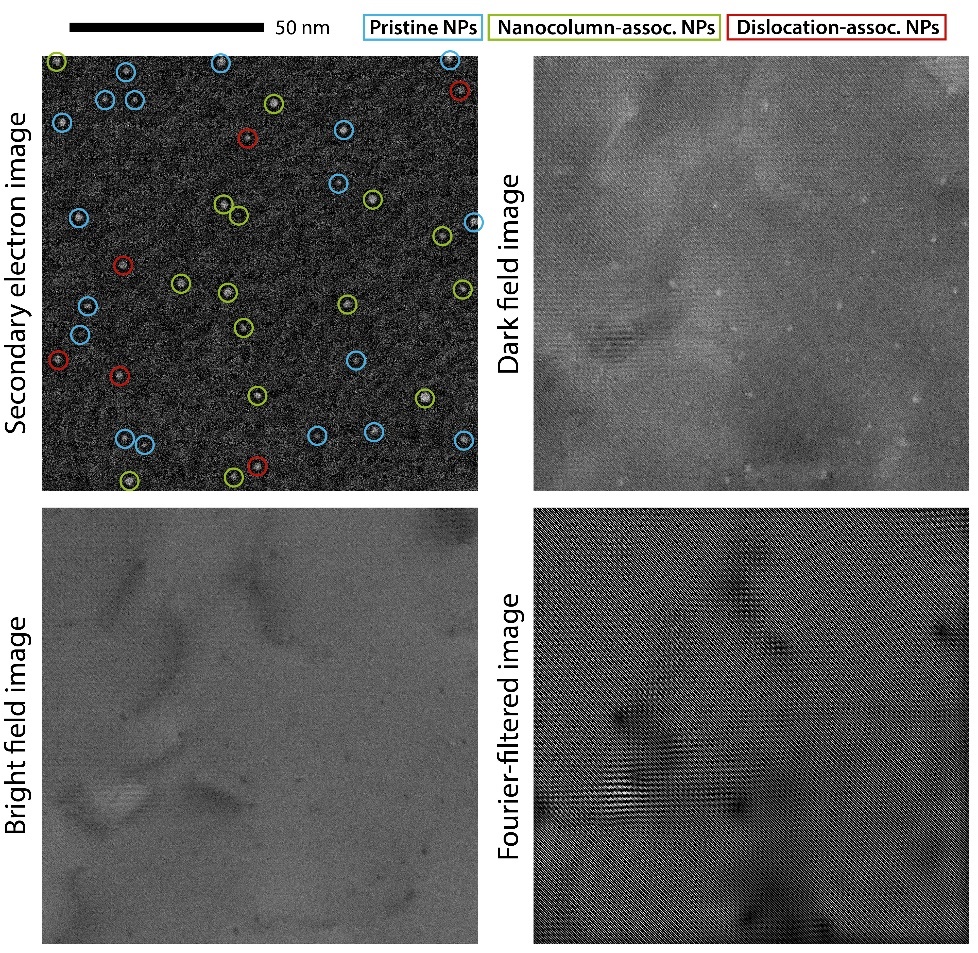


Figure S10. Correlated secondary electron, dark field and bright field imaging and well as Fourier transform. Region 1/6 used for a semi-quantitative statistical evaluation of the nanoparticle-support interface properties.


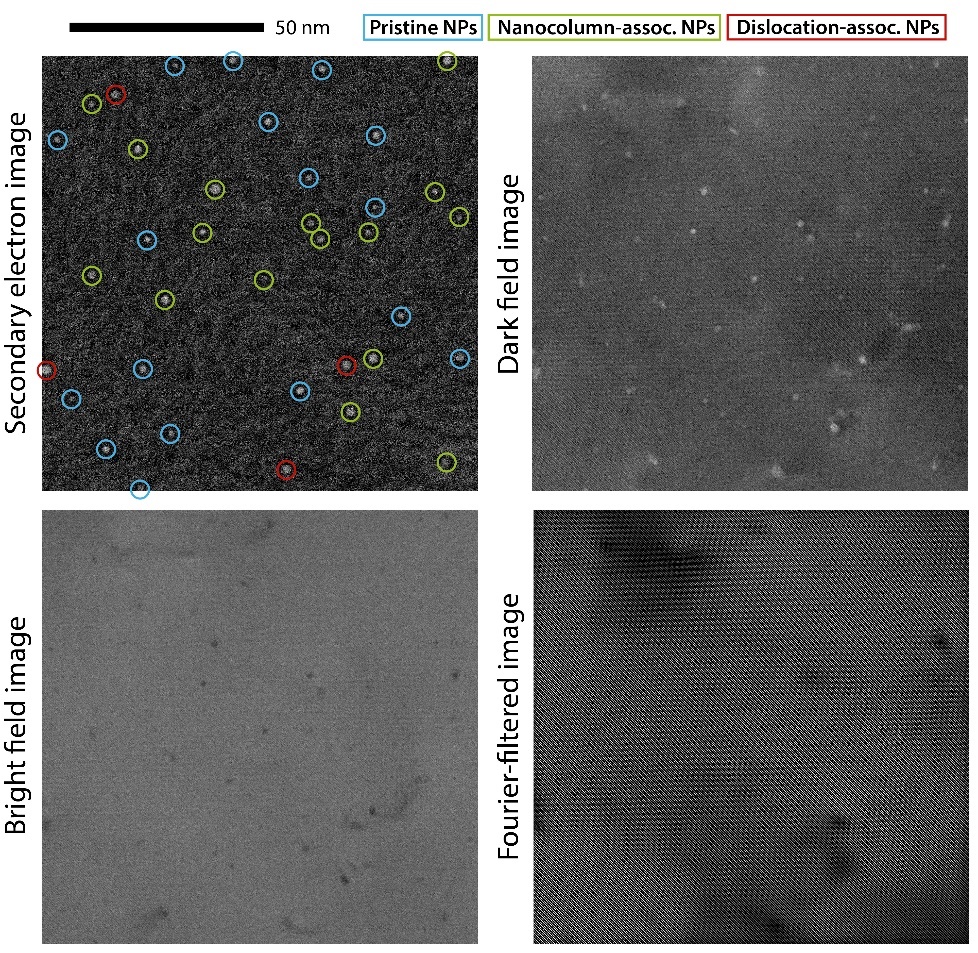


Figure S11. Correlated secondary electron, dark field and bright field imaging and well as Fourier transform. Region 2/6 used for a semi-quantitative statistical evaluation of the nanoparticle-support interface properties.


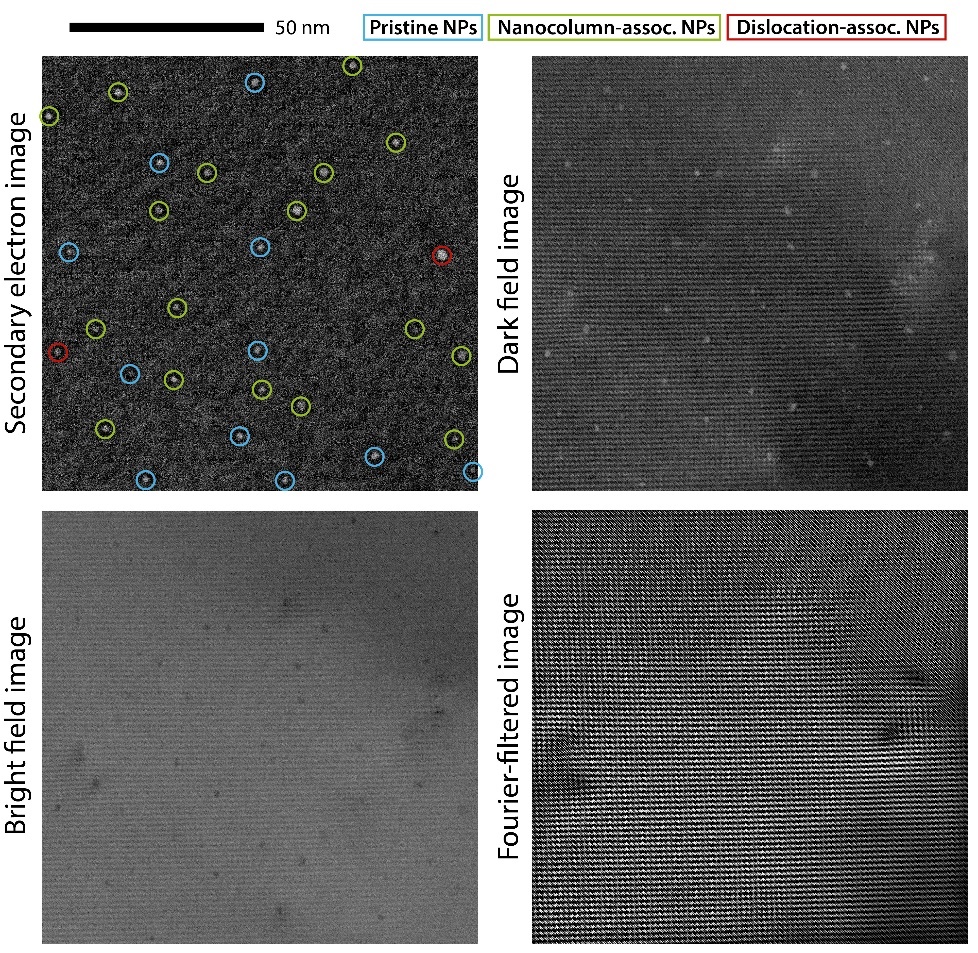


Figure S12. Correlated secondary electron, dark field and bright field imaging and well as Fourier transform. Region 3/6 used for a semi-quantitative statistical evaluation of the nanoparticle-support interface properties.


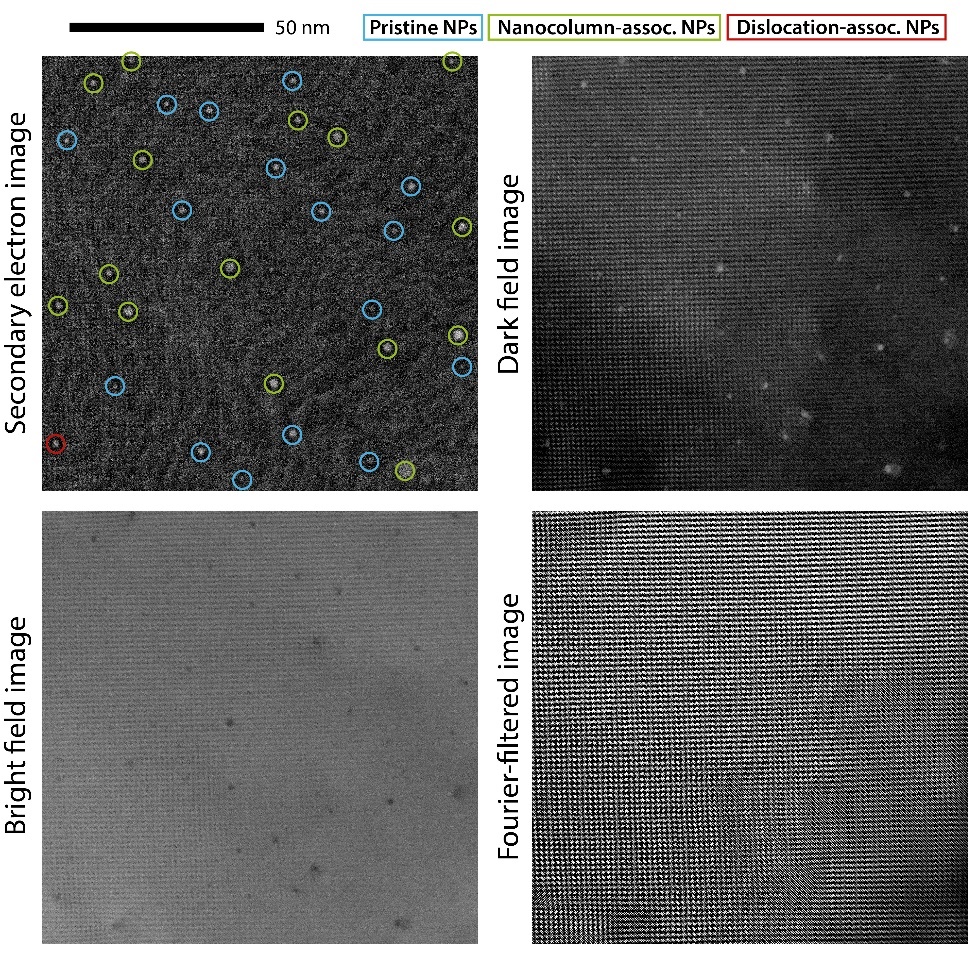


Figure S13. Correlated secondary electron, dark field and bright field imaging and well as Fourier transform. Region 4/6 used for a semi-quantitative statistical evaluation of the nanoparticle-support interface properties.


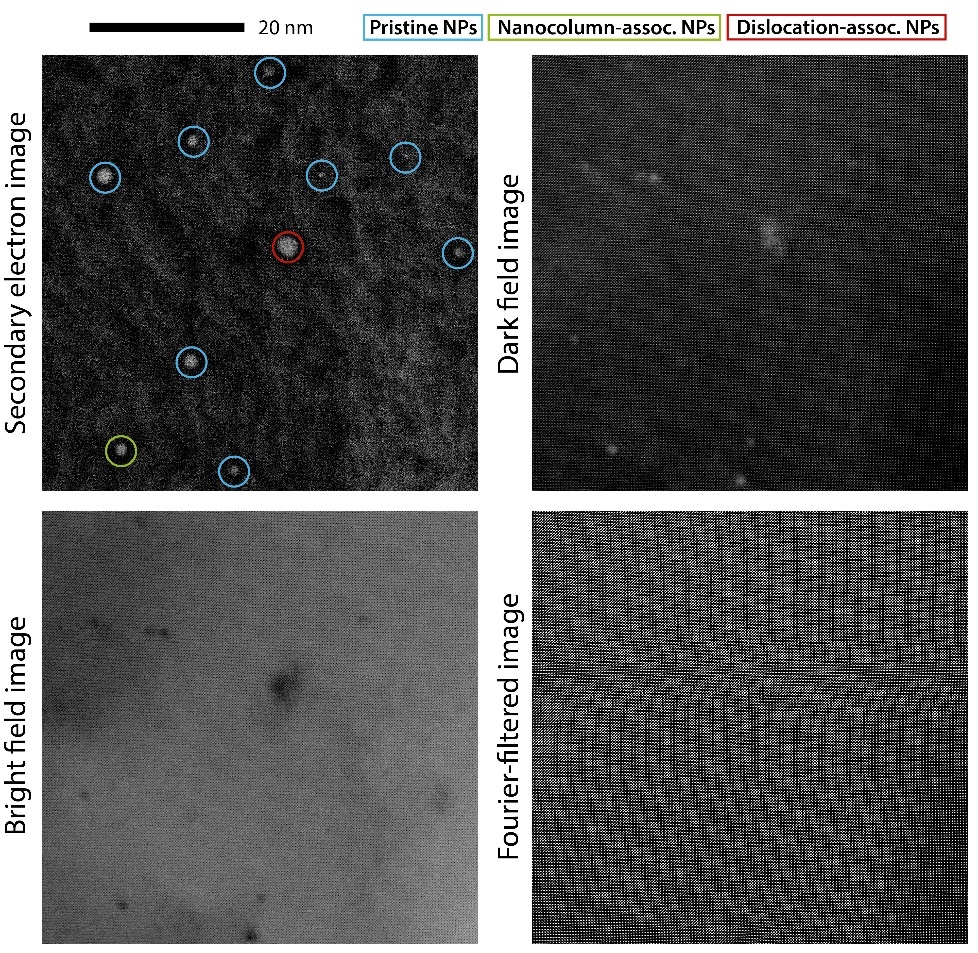


Figure S14. Correlated secondary electron, dark field and bright field imaging and well as Fourier transform. Region 5/6 used for a semi-quantitative statistical evaluation of the nanoparticle-support interface properties.


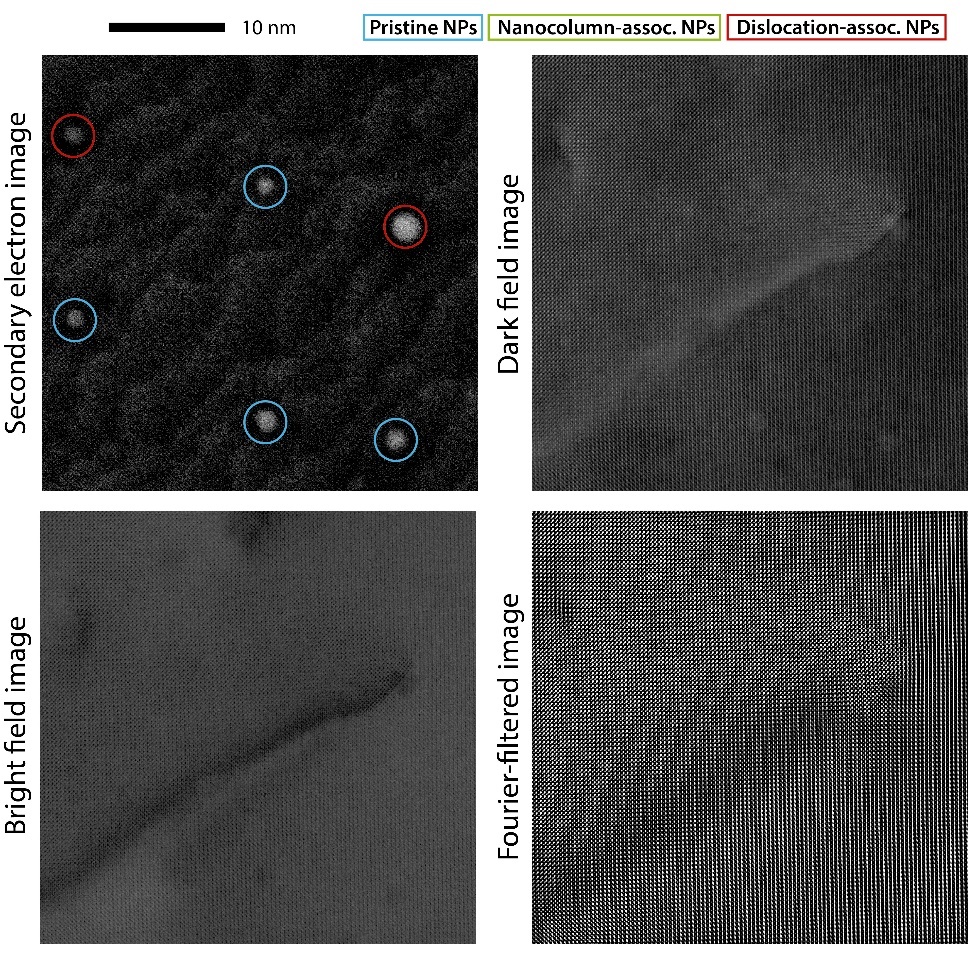


Figure S15. Correlated secondary electron, dark field and bright field imaging and well as Fourier transform. Region 6/6 used for a semi-quantitative statistical evaluation of the nanoparticle-support interface properties.

STEM images of lower magnification are used for a statistical evaluation of the nanoparticle-support interface characteristics. Based on this analysis, we provide a rough estimate of relative frequencies for exsolved nanoparticles that are sitting on the pristine perovskite oxide surface (not associated to defects), for nanocolumn-associated^[35]^ nanoparticles and for dislocation-associated nanoparticles.

For this purpose, contrast features in secondary electron images are compared to bright-field and dark-field images recorded from the same sample region. To make dislocations readily visible inverse fast-Fourier transformation (Fourier-filtered images) are obtained from the data, where lattice regions of low structural coherency appear as dark contrast features. Ni-enriched phase-separated defects become visible as bright in dark-field images. Note that both nanocolumn defects that are associated with exsolved nanoparticles at the surface and nanocolumn defects that remain buried in the oxide bulk without being associated with exsolved nanoparticles at the oxide surface are visible.

A total of 154 exsolved nanoparticles have been analyzed, as summarized in Figure S10 – Figure S15. Here, ~10% of the nanoparticles appear to be associated to dislocations in our sample, ~42% of the nanoparticles appear to be associated to nanocolumn defects and ~48% sit on the pristine perovskite oxide surface with no detectable defect present. Moreover, we estimate that ~60% of all dislocations investigated are associated with exsolved nanoparticles. Notably, the given nanoparticle distribution only reflects a snapshot, specifically describing our sample after a multi-step annealing procedure involving different annealing temperatures, annealing times and different annealing atmospheres. As we have demonstrated in our previous work, the surface processes under annealing conditions are typically highly dynamic^[35,37]^, where relative shares of nanoparticles associated to defects may differ significantly across different annealing conditions, and / or over annealing time. In this context, it is important to consider that the altered dislocation density may considerably change the exsolution and coalescence behavior of exsolved nanoparticles independent from the accumulation of Ni dopants at the dislocation cores and the impact on the energy barrier for nucleation. The reason is that dislocations in acceptor-doped SrTiO_3_ are surrounded by space charge tubes, as dictated by the global charge neutrality condition. In such space-charge tubes, oxygen vacancies are depleted. Given the fact that high oxygen vacancy concentrations at the nanoparticle-support interface will result in a decreased thermal stability of exsolved nanoparticles, the presence of potentially overlapping space-charge tubes of lower oxygen vacancy concentration may result in an increased stability of exsolved nanoparticles in the dislocation-engineered samples.


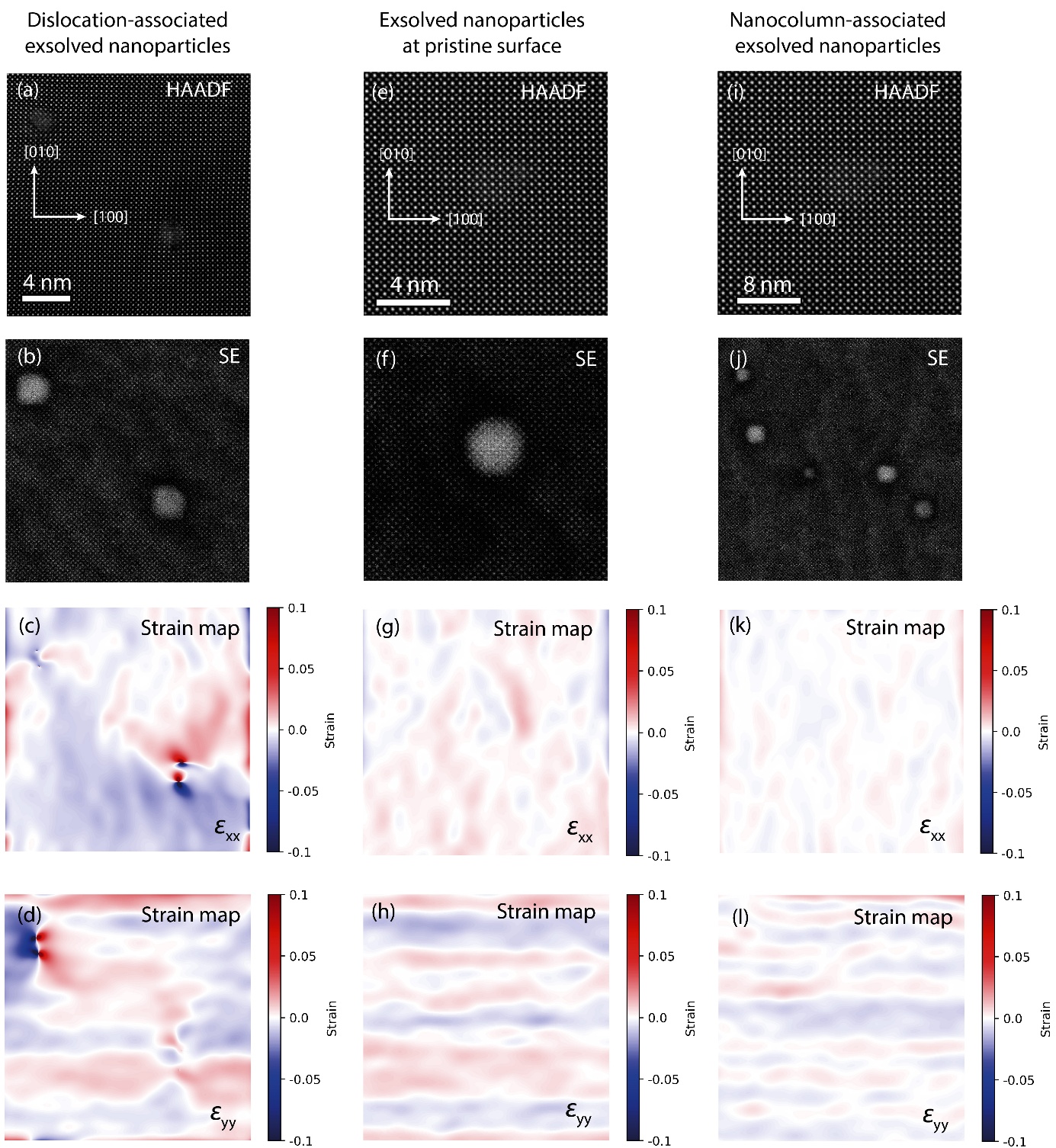


Figure S16. Correlated HAADF and SE imaging of exsolved nanoparticles and corresponding strain maps obtained by geometric phase analysis.


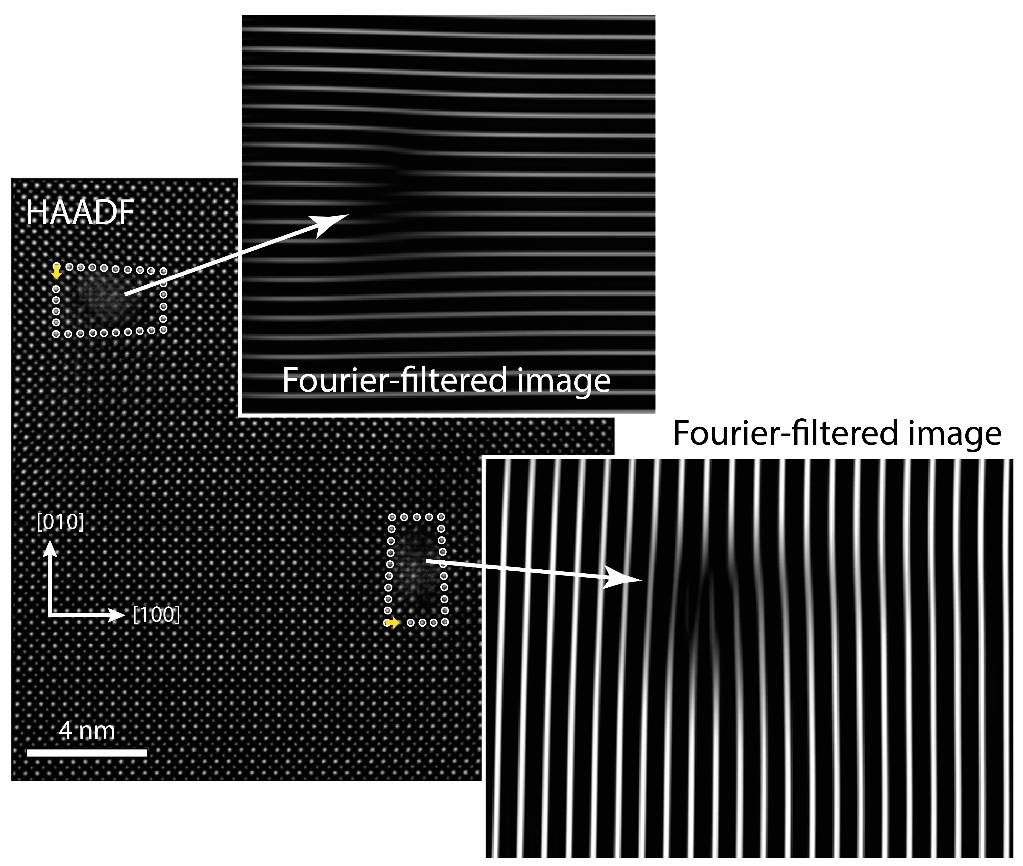


Figure S17. Fast-Fourier transforms obtained in the vicinity of two dislocation cores, indicating partial dissociation of the engineered dislocations.

References

[1] L. Porz, A. J. Klomp, X. Fang, N. Li, C. Yildirim, C. Detlefs, E. Bruder, M. Höfling, W. Rheinheimer, E. A. Patterson, P. Gao, K. Durst, A. Nakamura, K. Albe, H. Simons, J. Rödel, *Materials horizons* **2021**, *8*, 1528.

[2] X. Fang, *J American Ceramic Society* **2024**, *107*, 1425.

[3] D. N. Mueller, M. L. Machala, H. Bluhm, W. C. Chueh, *Nature communications* **2015**, *6*, 6097.

[4] J. T. Mefford, X. Rong, A. M. Abakumov, W. G. Hardin, S. Dai, A. M. Kolpak, K. P. Johnston, K. J. Stevenson, *Nature communications* **2016**, *7*, 11053.

[5] Z. Yin, H. Zhang, Y. Wang, Y. Wu, Y. Xing, X. Wang, X. Fang, Y. Yu, X. Guo, *Adv. Energy Mater.* **2024**.

[6] J. Kilner, *Solid State Ionics* **2000**, *129*, 13.

[7] K. K. Adepalli, J. Yang, J. Maier, H. L. Tuller, B. Yildiz, *Adv. Funct. Mater.* **2017**, *27*.

[8] A. Nakamura, K. Matsunaga, J. Tohma, T. Yamamoto, Y. Ikuhara, *Nature Mater* **2003**, *2*, 453.

[9] F. Gunkel, D. V. Christensen, Y. Z. Chen, N. Pryds, *Appl. Phys. Lett.* **2020**, *116*.

[10] F. Gunkel, D. V. Christensen, N. Pryds, *J. Mater. Chem. C* **2020**, *8*, 11354.

[11] S.-Y. Chung, S.-Y. Choi, H.-I. Yoon, H.-S. Kim, H. B. Bae, *Angewandte Chemie (International ed. in English)* **2016**, *55*, 9680.

[12] R. A. de Souza, F. Gunkel, S. Hoffmann-Eifert, R. Dittmann, *Phys. Rev. B* **2014**, *89*.

[13] R. Meyer, A. F. Zurhelle, R. A. de Souza, R. Waser, F. Gunkel, *Phys. Rev. B* **2016**, *94*.

[14] F. Gunkel, R. Waser, A. H. H. Ramadan, R. A. de Souza, S. Hoffmann-Eifert, R. Dittmann, *Phys. Rev. B* **2016**, *93*.

[15] Y.-M. Chiang, T. Takagi, *J American Ceramic Society* **1990**, *73*, 3278.

[16] H.-I. Yoon, D.-K. Lee, H. B. Bae, G.-Y. Jo, H.-S. Chung, J.-G. Kim, S.-J. L. Kang, S.-Y. Chung, *Nature communications* **2017**, *8*, 1417.

[17] R. A. de Souza, *Physical chemistry chemical physics PCCP* **2009**, *11*, 9939.

[18] R. Waser, *Solid State Ionics* **1995**, *75*, 89.

[19] D. Marrocchelli, L. Sun, B. Yildiz, *Journal of the American Chemical Society* **2015**, *137*, 4735.

[20] V. Metlenko, A. H. H. Ramadan, F. Gunkel, H. Du, H. Schraknepper, S. Hoffmann-Eifert, R. Dittmann, R. Waser, R. A. de Souza, *Nanoscale* **2014**, *6*, 12864.

[21] P. Kofstad, *Oxid Met* **1995**, *44*, 3.

[22] D. J. Keeble, S. Wicklein, R. Dittmann, L. Ravelli, R. A. Mackie, W. Egger, *Physical review letters* **2010**, *105*, 226102.

[23] Z. Wang, X. Hao, S. Gerhold, M. Schmid, C. Franchini, U. Diebold, *Phys. Rev. B* **2014**, *90*.

[24] M. L. Weber, M. Wilhelm, L. Jin, U. Breuer, R. Dittmann, R. Waser, O. Guillon, C. Lenser, F. Gunkel, *ACS nano* **2021**, *15*, 4546.

[25] M. B. Katz, S. Zhang, Y. Duan, H. Wang, M. Fang, K. Zhang, B. Li, G. W. Graham, X. Pan, *Journal of Catalysis* **2012**, *293*, 145.

[26] K. Syed, J. Wang, B. Yildiz, W. J. Bowman, *Nanoscale* **2022**, *14*, 663.

[27] K. Kousi, D. Neagu, L. Bekris, E. I. Papaioannou, I. S. Metcalfe, *Angewandte Chemie (International ed. in English)* **2020**, *59*, 2510.

[28] A. S. ARICÒ, P. BRUCE, B. SCROSATI, J.-M. TARASCON, W. van SCHALKWIJK, *Nature materials* **2005**, *4*, 366.

[29] H. Mistry, A. S. Varela, S. Kühl, P. Strasser, B. R. Cuenya, *Nat Rev Mater* **2016**, *1*.

[30] D. Neagu, G. Tsekouras, D. N. Miller, H. Ménard, J. T. S. Irvine, *Nature chemistry* **2013**, *5*, 916.

[31] Y. Gao, Z. Lu, T. L. You, J. Wang, L. Xie, J. He, F. Ciucci, *The journal of physical chemistry letters* **2018**, *9*, 3772.

[32] M. L. Weber, B. Šmíd, U. Breuer, M.-A. Rose, N. H. Menzler, R. Dittmann, R. Waser, O. Guillon, F. Gunkel, C. Lenser, *Nature Mater* **2024**, *23*, 406.

[33] H. Han, Y. Xing, B. Park, D. I. Bazhanov, Y. Jin, J. T. S. Irvine, J. Lee, S. H. Oh, *Nature communications* **2022**, *13*, 6682.

[34] J. Wang, J. Yang, A. K. Opitz, W. Bowman, R. Bliem, G. Dimitrakopoulos, A. Nenning, I. Waluyo, A. Hunt, J.-J. Gallet, B. Yildiz, *Chem. Mater.* **2021**, *33*, 5021.

[35] D. Jennings, M. L. Weber, A. Meise, T. Binninger, C. Price, M. Kindelmann, I. Reimanis, H. Matsumoto, P. Cao, R. Dittman, P. Kowalski, M. Heggen, O. Guillon, J. Mayer, F. Gunkel, W. Rheinheimer, *Direct Atomic-Scale Investigation of the Coarsening Mechanisms of Exsolved Catalytic Nanoparticles* **2024**.

[36] S. Singh, E. Prestat, L.-F. Huang, J. M. Rondinelli, S. J. Haigh, B. A. Rosen, *Scientific reports* **2017**, *7*, 10080.

[37] M. L. Weber, D. Jennings, S. Fearn, A. Cavallaro, M. Prochazka, A. Gutsche, L. Heymann, J. Guo, L. Yasin, S. J. Cooper, J. Mayer, W. Rheinheimer, R. Dittmann, R. Waser, O. Guillon, C. Lenser, S. J. Skinner, A. Aguadero, S. Nemšák, F. Gunkel, *Nat Commun* **2024**, *15*.

[38] Xufei Fang, Atsutomo Nakamura, Jürgen Rödel, *ACerS Bulletin* **2023**.

[39] H. Kim, S. Choi, P. Guha, H. Kim, J. Kim, S. Kim, R. Harder, W. Cha, H. Suh, J. Ryu, S. Yang, H.-I. Ji, D.-H. Kwon, *Dislocations govern noble metal exsolution in perovskite oxide* **2024**.

[40] X. Fang, O. Preuß, P. Breckner, J. Zhang, W. Lu, *J American Ceramic Society* **2023**, *106*, 4540.

[41] D. Hull, D. J. Bacon, *Introduction to Dislocations*, Elsevier **2011**.

[42] H. Han, J. Park, S. Y. Nam, K. J. Kim, G. M. Choi, S. S. P. Parkin, H. M. Jang, J. T. S. Irvine, *Nature communications* **2019**, *10*, 1471.

[43] K. Takehara, Y. Sato, T. Tohei, N. Shibata, Y. Ikuhara, *J Mater Sci* **2014**, *49*, 3962.

[44] P. Gao, R. Ishikawa, B. Feng, A. Kumamoto, N. Shibata, Y. Ikuhara, *Ultramicroscopy* **2018**, *184*, 217.

[45] H. Du, C.-L. Jia, L. Houben, V. Metlenko, R. A. de Souza, R. Waser, J. Mayer, *Acta Materialia* **2015**, *89*, 344.

[46] Z. Zhang, W. Sigle, M. Rühle, *Physical review. B, Condensed matter* **2002**, *66*.

[47] C. L. Jia, A. Thust, K. Urban, *Phys. Rev. Lett.* **2005**, *95*, 225506.

[48] R. A. de Souza, V. Metlenko, D. Park, T. E. Weirich, *Phys. Rev. B* **2012**, *85*.

[49] W. Lee, J. W. Han, Y. Chen, Z. Cai, B. Yildiz, *Journal of the American Chemical Society* **2013**, *135*, 7909.

[50] D. Jennings, M. P. Zahler, Di Wang, Q. Ma, W. Deibert, M. Kindelmann, C. Kübel, S. Baumann, O. Guillon, J. Mayer, W. Rheinheimer, *Acta Materialia* **2024**, *273*, 119941.

[51] C. Yang, B. Feng, J. Wei, N. Shibata, Y. Ikuhara, *Journal of Materials Science & Technology* **2024**, *181*, 58.

[52] C. Hu, R. Dingreville, B. L. Boyce, *Computational Materials Science* **2024**, *232*, 112596.

[53] S. Y. Hu, L. Q. Chen, *Acta Materialia* **2001**, *49*, 463.

[54] G. Sánchez‐Santolino, J. Salafranca, S. T. Pantelides, S. J. Pennycook, C. León, M. Varela, *Phys. Stat. Sol. (a)* **2018**, *215*.

[55] J. Zamudio-García, F. Chiabrera, A. Morin-Martínez, I. E. Castelli, E. R. Losilla, D. Marrero-López, V. Esposito, *Nat Commun* **2024**, *15*, 8961.

[56] M. Kindelmann, I. Povsturgar, S. Kuffer, D. Jennings, J. N. Ebert, M. L. Weber, M. P. Zahler, S. Escolantico, L. Almar, J. M. Serra, P. Kaghazchi, M. Bram, W. Rheinheimer, J. Mayer, O. Guillon, *Controlling grain boundary segregation to tune the conductivity of ceramic proton conductors* **2024**.

[57] H. Tuller, *Solid State Ionics* **2000**, *131*, 143.

[58] C. Okafor, K. Ding, X. Zhou, K. Durst, J. Rödel, X. Fang, *J American Ceramic Society* **2022**, *105*, 2399.

[59] L. Jin, X. Guo, C. L. Jia, *Ultramicroscopy* **2013**, *134*, 77.

[60] X. Fang, W. Lu, J. Zhang, C. Minnert, J. Hou, S. Bruns, U. Kunz, A. Nakamura, K. Durst, J. Rödel, *Harvesting room-temperature plasticity in ceramics by mechanically seeded dislocations* **2024**.

[61] H. Schraknepper, T. E. Weirich, R. A. de Souza, *Physical chemistry chemical physics PCCP* **2018**, *20*, 15455.

[62] Z. Zhang, W. Sigle, W. Kurtz, M. Rühle, *Physical review. B, Condensed matter* **2002**, *66*.

[63] S.-Y. Choi, S.-D. Kim, M. Choi, H.-S. Lee, J. Ryu, N. Shibata, T. Mizoguchi, E. Tochigi, T. Yamamoto, S.-J. L. Kang, Y. Ikuhara, *Nano Lett.* **2015**, *15*, 4129.

[64] M. Santaya, C. E. Jiménez, M. D. Arce, E. A. Carbonio, L. M. Toscani, R. Garcia-Diez, A. Knop-Gericke, L. V. Mogni, M. Bär, H. E. Troiani, *International Journal of Hydrogen Energy* **2023**, *48*, 38842.

[65] J. H. Kim, J. K. Kim, J. Liu, A. Curcio, J.-S. Jang, I.-D. Kim, F. Ciucci, W. Jung, *ACS nano* **2021**, *15*, 81.

[66] A. Schwiers, D. Röhrer, C. Lenser, B. Steinrücken, D. Sebold, H. Spliethoff, O. Guillon, N. H. Menzler, *J. Mater. Chem. A* **2024**, *12*, 9132.

[67] Z. Shang, J. Zhang, L. Ye, K. Xie, *J. Mater. Chem. A* **2022**, *10*, 12458.

[68] A. López-García, S. Remiro-Buenamañana, D. Neagu, A. J. Carrillo, J. M. Serra, *Small (Weinheim an der Bergstrasse, Germany)* **2024**.

[69] A. López-García, L. Almar, S. Escolástico, A. B. Hungría, A. J. Carrillo, J. M. Serra, *ACS Appl. Energy Mater.* **2022**, *5*, 13269.

[70] H. Qi, T. Yang, W. Li, L. Ma, S. Hu, W. Shi, E. M. Sabolsky, J. W. Zondlo, R. Hart, G. A. Hackett, X. Liu, *ECS Trans.* **2019**, *91*, 1701.

[71] P. Cao, P. Tang, M. F. Bekheet, H. Du, L. Yang, L. Haug, A. Gili, B. Bischoff, A. Gurlo, M. Kunz, R. E. Dunin-Borkowski, S. Penner, M. Heggen, *J. Phys. Chem. C* **2022**, *126*, 786.

[72] L. Aballe, S. Matencio, M. Foerster, E. Barrena, F. Sánchez, J. Fontcuberta, C. Ocal, *Chem. Mater.* **2015**, *27*, 6198.

[73] Y.-R. Jo, B. Koo, M.-J. Seo, J. K. Kim, S. Lee, K. Kim, J. W. Han, W. Jung, B.-J. Kim, *Journal of the American Chemical Society* **2019**, *141*, 6690.

[74] N. W. Kwak, S. J. Jeong, H. G. Seo, S. Lee, Y. Kim, J. K. Kim, P. Byeon, S.-Y. Chung, W. Jung, *Nature communications* **2018**, *9*, 4829.

[75] J. Wang, D. Kalaev, J. Yang, I. Waluyo, A. Hunt, J. T. Sadowski, H. L. Tuller, B. Yildiz, *Journal of the American Chemical Society* **2023**, *145*, 1714.

[76] R. A. de Souza, *Current Opinion in Solid State and Materials Science* **2021**, *25*, 100923.

[77] W. H. RHODES, W. D. KINGERY, *J American Ceramic Society* **1966**, *49*, 521.

[78] Y. H. Kim, H. Jeong, B.-R. Won, J.-H. Myung, *Adv. Mater.* **2023**, *35*, e2208984.

[79] W. Rheinheimer, X. L. Phuah, L. Porz, M. Scherer, J. Cho, H. Wang, *Journal of the European Ceramic Society* **2023**, *43*, 3524.

[80] D. Neagu, T.-S. Oh, D. N. Miller, H. Ménard, S. M. Bukhari, S. R. Gamble, R. J. Gorte, J. M. Vohs, J. T. S. Irvine, *Nature communications* **2015**, *6*, 8120.

[81] M. Wang, E. I. Papaioannou, I. S. Metcalfe, A. Naden, C. D. Savaniu, J. T. S. Irvine, *Adv. Funct. Mater.* **2023**, *33*.

[82] D. Neagu, E. I. Papaioannou, W. K. W. Ramli, D. N. Miller, B. J. Murdoch, H. Ménard, A. Umar, A. J. Barlow, P. J. Cumpson, J. T. S. Irvine, I. S. Metcalfe, *Nature communications* **2017**, *8*, 1855.

[83] R. Wang, Y. Zhu, S. M. Shapiro, *Phys. Rev. Lett.* **1998**, *80*, 2370.

[84] Francisco de la Peña, Eric Prestat, Vidar Tonaas Fauske, Pierre Burdet, Jonas Lähnemann, Petras Jokubauskas, Tom Furnival, Magnus Nord, Tomas Ostasevicius, Katherine E. MacArthur, Duncan N. Johnstone, Mike Sarahan, Joshua Taillon, Thomas Aarholt, pquinn-dls, Vadim Migunov, Alberto Eljarrat, Jan Caron, Carter Francis, T. Nemoto, Timothy Poon, Stefano Mazzucco, actions-user, Nicolas Tappy, Niels Cautaerts, Suhas Somnath, Tom Slater, Michael Walls, Florian Winkler, Håkon Wiik Ånes, *hyperspy/hyperspy: Release v1.7.2*, Zenodo **2022**.

[85] H. Sternlicht, S. A. Bojarski, G. S. Rohrer, W. D. Kaplan, *Journal of the European Ceramic Society* **2018**, *38*, 1829.

[86] *GPA-geometrical phase analysis software* **2018**.
